# Probing Anti-Leukemic Metabolites from Marine-Derived *Streptomyces* sp. LY1209

**DOI:** 10.3390/metabo12040320

**Published:** 2022-04-02

**Authors:** You-Ying Chen, Lo-Yun Chen, Po-Jen Chen, Mohamed El-Shazly, Bo-Rong Peng, Yu-Cheng Chen, Chun-Han Su, Jui-Hsin Su, Ping-Jyun Sung, Pei-Tzu Yen, Lung-Shuo Wang, Kuei-Hung Lai

**Affiliations:** 1 Department of Marine Biotechnology and Resources, National Sun Yat-Sen University, Kaohsiung City 80424, Taiwan; zoeblack0108@gmail.com (Y.-Y.C.); x2219@nmmba.gov.tw (J.-H.S.); pjsung@nmmba.gov.tw (P.-J.S.); 2 Graduate Institute of Pharmacognosy, College of Pharmacy, Taipei Medical University, Taipei 11031, Taiwan; m303110004@tmu.edu.tw; 3 Department of Medical Research, E-Da Hospital, Kaohsiung City 82445, Taiwan; ed113510@edah.org.tw; 4 Department of Pharmacognosy, Faculty of Pharmacy, Ain-Shams University, Organization of African Unity Street, Abassia, Cairo 11566, Egypt; mohamed.elshazly@pharma.asu.edu.eg; 5 Department of Pharmaceutical Biology, German University in Cairo, Cairo 11432, Egypt; 6 National Museum of Marine Biology & Aquarium, Pingtung 94450, Taiwan; pengpojung@gmail.com; 7 Sepsis Research Center, Research Center of Tropical Medicine and Infectious Disease, Graduate Institute of Medicine, School of Medicine, Kaohsiung Medical University, Kaohsiung 80708, Taiwan; r080195@kmu.edu.tw; 8 Department of Food Science, College of Human Ecology, Fu Jen Catholic University, New Taipei City 24205, Taiwan; 154286@mail.fju.edu.tw; 9 Ph.D. Program in Pharmaceutical Biotechnology, Fu Jen Catholic University, New Taipei City 24205, Taiwan; 10 Department of Chinese Medicine, Sin-Lau Hospital, Tainan 70142, Taiwan; agneskyetabo@gmail.com; 11 The School of Chinese Medicine for Post Baccalaureate, I-Shou University, Kaohsiung 82445, Taiwan; 12 Ph.D. Program in Clinical Drug Development of Herbal Medicine, College of Pharmacy, Taipei Medical University, Taipei 11031, Taiwan; 13 Traditional Herbal Medicine Research Center, Taipei Medical University Hospital, Taipei 11031, Taiwan

**Keywords:** *Streptomyces* sp. LY1209, chromatographic bioassay profile, staurosporine, diketopiperazines

## Abstract

The unmet need for specific anti-leukemic agents for the treatment of acute lymphoblastic leukemia led us to screen a variety of marine-derived bacteria. The fermentation broth extract of *Streptomyces* sp. LY1209 exhibited the most potent anti-proliferative effect against Molt 4 leukemia cells. A chromatographic anti-proliferative profiling approach was applied to characterize the metabolites with bioactive potential. Among all the metabolites, the major anti-leukemic constituents were staurosporine and a series of diketopiperazines (DKPs), including one novel and two known DKPs identified from nature for the first time. The structures of these compounds were identified using extensive spectroscopic analysis. The anti-proliferative potential of these metabolites against the Molt 4 cancer cell line was also determined. According to the in silico analysis utilizing a chemical global positioning system for natural products (ChemGPS-NP), it was suggested that these DKPs are potential anti-microtubule and alkylating agents, while staurosporine was proposed to be a tyrosine kinase inhibitor. Our findings not only identified a series of anti-proliferative metabolites, but also suggested a strategic workflow for the future discovery of natural product drug leads.

## 1. Introduction

Acute lymphocytic leukemia (ALL) is invasive leukemia characterized by the proliferation of lymphoblasts or lymphocytes in the bone marrow and peripheral blood. These immature leukemic cells can spread to the lymph nodes, spleen, liver, central nervous system, and other organs. They can displace normal red blood cells [1]. ALL progresses quickly if not treated, and it causes thousands of deaths annually in the United States, according to the American Cancer Society. ALL patients are usually treated with a combination of unspecific conventional chemotherapeutic agents such as vincristine, imatinib, etoposide, methotrexate, and cytarabine [2]. Despite the diversity of the drugs used, serious side effects are the most common drawbacks of these chemotherapeutic regimens. In addition, the use of common cytotoxic drugs for ALL urges the scientific community to search for more specific agents [3,4]. The Molt 4 cell line is the most sensitive T-cell ALL cell line, and can be used to search for potential novel anti-leukemic agents.

*Streptomyces* sp. bacteria have drawn scientists’ attention for several decades since the discovery of the first antibiotic, streptomycin, from *Streptomyces griseus* in 1943 [5]. The subsequent chemical and pharmacological investigation of *Streptomyces* sp. led to the discovery of several clinical chemotherapeutic agents, such as dactinomycin, doxorubicin, daunorubicin, and anthracyclines. Insight into these interesting secondary metabolites, diketopiperazines (DKPs, also called dipeptides, dipeptide anhydrides, or 2,5-dioxopiperazines), a group of compounds identified from *Streptomyces* sp., drew much attention due to their well-known activity as antibacterial, anti-proliferative, anti-hepatoma, and anti-fungal agents [6,7]. A highly oxidized DKP, bicyclomycin, was identified from *Streptomyces cinnamoneus* and was approved as an antibiotic with an unusual mechanism of action [8]. Several recent studies have focused on discovering the cytotoxic potential of these DKPs [9,10,11,12,13,14], but relatively few research efforts have addressed their anti-leukemic properties [15]. The rich chemical diversity and potent cytotoxic potential of *Streptomyces* sp. motivated us to screen a series of marine-derived strains of this genus. In the current study, we aimed to illustrate the anti-proliferative potential of our identified strain, *Streptomyces* sp. LY1209, and to further examine its major bioindicators based on a chromatographic anti-proliferative profiling approach. Moreover, a computational tool based on the principal component analysis (PCA) of physical–chemical properties, ChemGPS-NP, was applied to interpret the possible mode of its anti-proliferative activity.

## 2. Results and Discussion

### 2.1. Establishment of High-Performance Liquid Chromatography (HPLC)-Based High-Resolution Anti-Proliferative Profile

**Figure 1:**
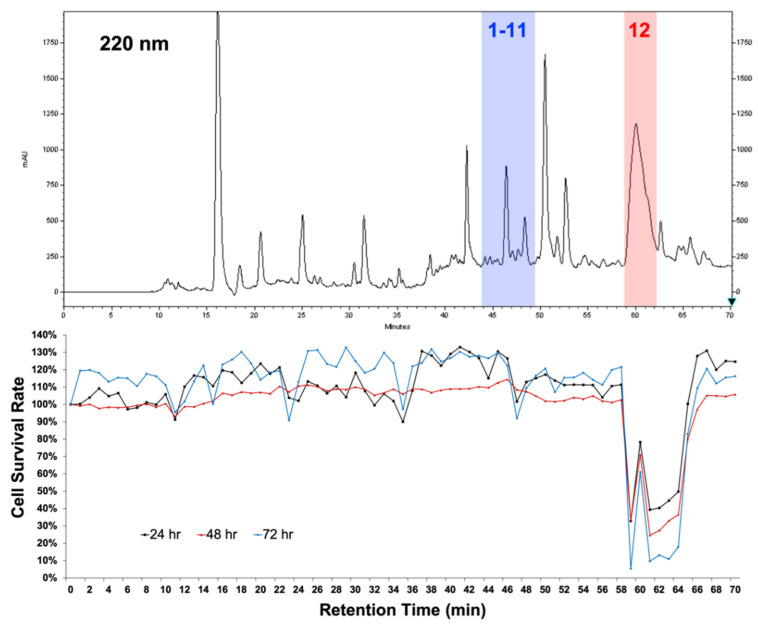
High-resolution anti-proliferative profile of the ethyl acetate (EtOAc) extract (1 μg/mL) of *Streptomyces* sp. LY1209 against Molt 4 cells.

**Figure 2:**
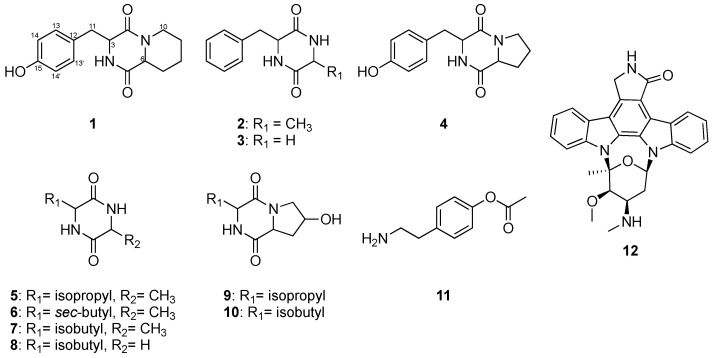
The identified compounds (**1**–**12**) from EtOAc extract of *Streptomyces* sp. LY1209.

Fractionation methods with traditional column chromatography cannot overcome the problem of overlapping secondary metabolites, which reduces the accuracy of the following bioassay-guided isolation step for the purification of pure compounds with biological activity. To overcome this problem, we used high-performance liquid chromatography (HPLC) to ensure the distinctive separation of the test fractions during bioassay. The chromatogram retention time (per 1 min)-based fractionation of an ethyl acetate (EtOAc) extract of Streptomyces sp. LY1209 was performed within 70 min. The collected fractions (at a concentration of 1 μg/mL) were further subjected to MTT cell proliferation assay to provide a high-resolution cell proliferative profile against Molt 4 cells (Figure 1). Among all test fractions, the 59th to 64th fractions (labeled with transparent red) exhibited the most potent activity. The corresponding tailing peak in the HPLC profile was identified as staurosporine (**12**) [16] using nuclear magnetic resonance (NMR) and mass (MS) spectroscopy. Other chromatographic peaks showing relatively less anti-proliferative activity, such as the 44th to 49th fractions (labeled with transparent blue), were also selected for scale-up production. Further separation and isolation were performed to yield eleven diketopiperazines (DKPs) (Figure 2), which were elucidated as 3-(4-hydroxybenzyl)-hexahydro-pyrido[1,2-a]pyrazine-1,4)-dione (**1**), cyclo-(Ala-Phe) (**2**) [17], cyclo-(Gly-Phe) (**3**) [18], cyclo-(Pro-Tyr) (**4**) [19], cyclo-(Ala-Val) (**5**) [20], cyclo-(Ile-Ala) (**6**) [21], cyclo-(Leu-Ala) (**7**) [22], cyclo-(Gly-Leu) (**8**) [23], (3S,7R,8aS)-7-hydroxy-3-isopropylhexahydropyrrolo[1,2-a]pyrazine-1,4-dione (**9**) [24], cyclo(Leu-hydroxy-Pro) (**10**) [23], and 4-(2-aminoethyl)-phenylacetate (**11**) [25].

### 2.2. Structure Elucidation of Diketopiperazines (DKPs) Obtained from Streptomyces sp. LY1209

**Figure 3:**
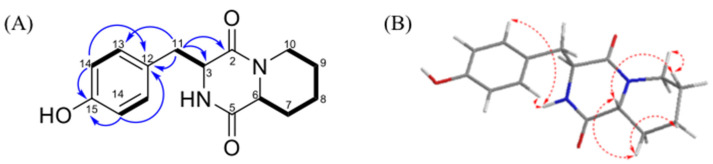
(**A**) The COSY and HMBC of **1**. (**B**) The NOESY correlations of **1**.

Compound **1** was obtained as a yellow amorphous solid. The molecular formula of **1** was revealed to be C_15_H_16_N_2_O_3_ based on the HRESIMS peak at *m*/*z* 275.1406 (calculated for C_15_H_1__9_N_2_O_3_, 275.1396, and ^13^C NMD data suggesting eight degrees of unsaturation). The IR spectrum revealed the presence of hydroxyls, carbonyls, benzene rings, and NH-bonded moieties. Based on the ^13^C NMR (in CD_3_OD) and DEPT spectroscopic data of **1** (Table 1), fifteen carbon signals were observed including five sp^3^ methylene groups (*δ*_C_ 24.8, 25.3, 31.6, 40.9, and 43.3); two sp^3^ methines (*δ*_C_ 57.2 and 58.4); four sp^2^ olefinic methines (*δ*_C_ 116.2 (two overlapped signals) and 132.5 (two overlapped signals)); and four quaternary carbons (*δ*_C_ 126.5, 158.2, 167.0, and 169.9). Among these NMR signals, two amide carbonyls at *δ*_C_ 167.0 (C) and 169.9 (C) were characterized, along with C-3 (57.2) and C-6 (58.4), suggesting a diketopiperazine skeleton [26]. This functionality was also supported by the HMBC cross-peaks (Figure 3) from H-3 (*δ*_H_ 4.23) to C-2 (*δ*_C_ 167.0) and to C-5 (*δ*_C_ 169.9). A 1,4-disubstituted phenyl group was suggested based on a pair of overlapping signals at *δ*_H_ 6.70 (2H, d, 8.5 Hz)/*δ*_C_ 116.2 (CH) and 6.91 (2H, d, 8.5 Hz)/*δ*_C_ 132.5 (CH). The connection between both moieties was determined according to the HMBC correlations from H-11 (*δ*_H_ 3.17) to C-3 (*δ*_C_ 57.2) and C-12 (*δ*_C_ 126.5). Furthermore, the elucidated phenyl diketopiperazine group accounted for seven of the total eight degrees of unsaturation, implying the presence of an additional ring. The assignment of the additional piperidine group was elucidated based on the COSY (Figure 3) correlations of H-6/H-7, H-7/H-8, H-7/H-9, H-8/H-9, and H-9/H-10. Thus, the planar structure of **1** was verified.

Based on the ^1^H NMR coupling constants of this type of compound [27], the α-face-H-6 and trans-ring junction of **1** were suggested from the coupling constant of H-6 (11.3, 1.6 Hz). The NOESY correlations between H-6/H-7α, H-6/H-10α, H-7α/H-8α, H-9α/H-10α indicated the same orientation of these protons, referring to the 6S of **1**. Additionally, the 3R configuration was elucidated based on the NOESY correlation of H-3/H-13, as well as the non-cross-peak between H-3 and H-6. Consequently, compound **1** was established and named as 3-(4-hydroxybenzyl)-hexahydro-pyrido[1,2-a]pyrazine-1,4)-dione.

Compounds **2**–**11** were identified by comparing their NMR data with those reported in previous literature (Figures S12–S46). The molecular weights of the identified compounds were also confirmed using MS spectrometry. Among these compounds, **9** and **10** were isolated from natural sources for the first time.

### 2.3. Anti-Proliferative Assessments of the Isolated Compounds

The biological activity of the isolated DKPs and the indolocarbazole against Molt 4 cell lines was evaluated using the MTT cell proliferation assay (Table 2). The results indicated the potent anti-proliferative activity of **12** (IC_50_: 0.01 μM). Among all the tested DKPs, only the derivative with 3-sec-butyl functionality exhibited moderate activity (IC_50_: 56.23 μM), suggesting the importance of this functionality for further medicinal modification. Therefore, staurosporine (**12**) was suggested to dominate the anti-proliferative activity of the EtOAc extract of *Streptomyces* sp. LY1209 against the Molt 4 cell.

### 2.4. Navigating Potential Therapeutical Targets of Anti-Proliferative Compounds Using ChemGPS-NP

**Figure 4:**
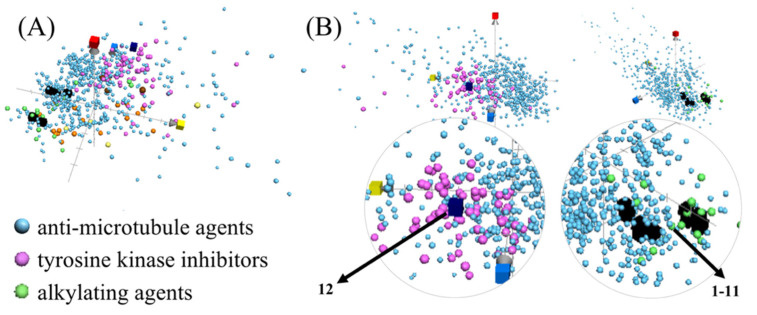
The plotted mode of action-relevant chemical patterns of the isolated compounds (DKPs **1**–**11** (black), and **12** (dark blue)), and the clinical chemotherapeutic agents searched from the ChEMBL database (alkylating agents (green, 19 compounds), anti-metabolites (orange, 21 compounds), proteasome inhibitors (yellow, 4 compounds), tyrosine kinase inhibitors (pink, 74 compounds), topoisomerase inhibitors (brown, 3 compounds), and anti-microtubule agents (light blue, 642 compounds)). The score plot was marked with three dimensions (principal components) including PC1 (red) describing the size, shape, and polarizability; PC2 (blue) representing the aromatic- and conjugation-related properties; and PC3 (yellow) depicting lipophilicity, polarity, and H-bond capacity. The overall view (**A**) and the individual views (**B**) of the analytical results.

Since the innovation of Lipinski’s rule-of-five, characterizing the drug-likeness of natural small molecules based on certain chemical and physical properties has become a promising approach for the development of potential lead compounds [28]. The recently developed principle component analysis (PCA)-based computational model, chemical global positioning system for natural products (ChemGPS-NP) [29], can assist in mapping the drug-likeness of compounds in virtual chemical space, and can further illustrate their potential pharmacological or biological targets [30,31,32,33,34,35,36,37]. ChemGPS-NP was applied to predict the possible therapeutic targets of our compounds compared with clinical chemotherapeutic agents with six prominent action modes, including alkylating agents, anti-metabolites, proteasome inhibitors, tyrosine kinase inhibitors, topoisomerase inhibitors, and anti-microtubule agents. The results (Figure 4) suggest that DKPs (labeled as black cubes) are potential anti-microtubule and alkylating agents, due to their similar location in the ChemGPS-NP chemical space with the corresponding clinical agents, while staurosporine was predicted to be a tyrosine kinase inhibitor.

The proposed mode of action of DKPs (**1**–**11**) was previously proven through the synthesis of DKPs with anti-microtubule [38,39] and alkylating [40,41] activities. The predicted mode of action of staurosporine (**12**) as a multi-targeted protein kinase inhibitor, is similar to that reported for a closely related derivative, midostaurin [42], suggesting the potential application of indolocarbazole derivatives for the treatment of ALL.

## 3. Materials and Methods

### 3.1. General Experimental Procedure

IR spectra were measured on a Fourier-transform IR spectrophotometer, Varian Digilab FTS 1000 (Varian Inc., Palo Alto, CA, USA). NMR spectroscopic analyses were performed using a Varian Unity INOVA 500 FT-NMR instrument (Varian Inc., Palo Alto, CA, USA). High-resolution electrospray ionization mass spectrometry (HRESIMS) analyses were carried out using a Bruker APEX II instrument (Bruker Daltonik, Bremen, Germany). All methods were carried out following the relevant guidelines and regulations.

### 3.2. Isolation, Culture, and Extraction of Marine Bacteria

Marine bacterium strain *Streptomyces* sp. LY1209 was isolated from benthic sediment, which was collected from the Kenting coast, south of Taiwan on 6 September 2012, at a depth of 2000 m. The bacterium strain LY1209 was 99% identical to *Streptomyces fradiae* (Genebank accession no. NR043485.1) based on the 16S rRNA gene sequence. The marine bacterium was cultured in 2.5 L flasks containing 1 L M1 broth (1% starch, 0.4% yeast extract, 0.2% peptone, without agar) with 80% seawater. The bacterial culture was incubated at 25 °C in an orbital shaker (120 rpm). The incubation was terminated after five days by adding ethyl acetate (EtOAc, 3 × 60 L) to extract the culture broth (60 L). The total obtained extract was 6.64 g.

### 3.3. Fraction Preparation for HPLC-Based High-Resolution Anti-Proliferative Assay

A Hitachi Elite LaChrom HPLC system (Hitachi, Tokyo, Japan) consisting of a Hitachi L-2130 pump and a Hitachi L-2455 Photodiode Array Detector was employed to profile the EtOAc extract of *Streptomyces* sp. LY1209. Liquid chromatography was performed using a COSMOSIL 5C18-MS-II Waters (20 × 250 mm, C_18_) column (Nacalai Tesque, Kyoto, Japan). The mobile phase was mixed with MeOH (M) and water (W). A gradient sequence was executed as follows: The initial eluting condition was M–W (10:90, *v*/*v*), linearly changed to M–W (30:70, *v*/*v*) at 25 min, M–W (45:55, *v*/*v*) at 30 min, then the final condition was M–W (80:20, *v*/*v*) at 70 min. The flow rate was set at 5 mL/min, the temperature of the column was maintained at 25 °C, and the detection wavelength was fixed at 220 nm. As for the dry EtOAc extract, 10 mg was dissolved in 100 μL of methanol and filtered through a 0.45 m membrane filter before loading onto the HPLC column. Then, sample injection was performed manually with 100 μL volume per injection. Fractionation yielded a total of 70 fractions, which were collected every 1 min according to the retention time. The solvents of the collected fractions were removed using a sample concentrator and vacuum evaporator. At a concentration of 1 μg/mL, all fractions were subjected to the final MTT cell proliferation assay to examine their anti-proliferative properties. The fractions with anti-proliferative potential were produced in larger quantities and were subjected to reversed-phase chromatographic isolation and purification. The purification of DKPs (**1**–**11**) from the 44th to 49th fractions was executed by isocratic elution (M–W (50:50, *v*/*v*)). The combination of the 59th to 64th fractions, obtained by gradient elution, yielded pure staurosporine (**12**). The identification of the isolated compounds was completed based on a comparison between the obtained spectroscopic data (nuclear magnetic resonance (NMR) and mass (MS) spectra) and the previously reported ones (please refer to the spectroscopic data in the Supplementary Materials).

3-(4-Hydroxybenzyl)-hexahydro-pyrido[1,2-a]pyrazine-1,4)-dione (**1**): Yellow powder; IR 3367, 1635 cm^−^^1^; UV-vis (MeOH) *λ*_max_ (log *ε*) 206, 219, 239, 278, 289 nm; ^1^H (500 MHz, CD_3_OD) and ^13^C (125 MHz, CD_3_OD) NMR spectroscopic data, Table 1; ESIMS *m*/*z* 481 [M + H]^+^; HRESIMS *m*/*z* 481.29259 [M + H]^+^ (calcd. for C_28_H42O5, 481.29245).

Cyclo-(Ala-Phe) (**2**): White needles, ^1^H (500 MHz, CD_3_OD) and ^13^C (125 MHz, CD3OD) NMR spectroscopic data; ESIMS *m*/*z* 219 [M + H]^+^.

Cyclo-(Gly-Phe) (**3**): White amorphous powder, ^1^H (500 MHz, CD_3_OD) and ^13^C (125 MHz, CD_3_OD) NMR spectroscopic data, Table S2; ESIMS *m*/*z* 205 [M + H]^+^.

Cyclo-(Pro-Tyr) (**4**): Yellow oil, ^1^H (500 MHz, CD_3_OD) and ^13^C (125 MHz, CD_3_OD) NMR spectroscopic data, Table S3; ESIMS *m*/*z* 261 [M + H]^+^.

Cyclo-(Ala-Val) (**5**): White powder, ^1^H (500 MHz, CD_3_OD) and ^13^C (125 MHz, CD_3_OD) NMR spectroscopic data, Table S4; ESIMS *m*/*z* 171 [M + H]^+^.

Cyclo-(Ile-Ala) (**6**): White powder, ^1^H (500 MHz, CD_3_OD) and ^13^C (125 MHz, CD_3_OD) NMR spectroscopic data, Table S5; ESIMS *m*/*z* 185 [M + H] ^+^.

Cyclo-(Leu-Ala) (**7**): white amorphous, ^1^H (500 MHz, CD_3_OD) and ^13^C (125 MHz, CD_3_OD) NMR spectroscopic data, Table S2; ESIMS *m*/*z* 185 [M + H]^+^.

Cyclo-(Gly-Leu) (**8**): White powder, ^1^H (500 MHz, CD_3_OD) and ^13^C (125 MHz, CD_3_OD) NMR spectroscopic data, Table S7; ESIMS *m*/*z* 171 [M + H]^+^.

(3*S*,7*R*,8a*S*)-7-Hydroxy-3-isopropylhexahydropyrrolo[1,2-a]pyrazine-1,4-dione (**9**): colorless oil, ^1^H (500 MHz, CD_3_OD) and ^13^C (125 MHz, CD_3_OD) NMR spectroscopic data, Table S8; ESIMS *m*/*z* 213 [M + H]^+^.

Cyclo(Leu-hydroxy-Pro) (**10**): Colorless crystals, ^1^H (500 MHz, CD_3_OD) and ^13^C (125 MHz, CD_3_OD) NMR spectroscopic data, Table S9.

4-(2-Aminoethyl)-phenyl acetate (**11**): White powder, ^1^H (500 MHz, CD_3_OD) and ^13^C (125 MHz, CD_3_OD) NMR spectroscopic data, Table S10; ESIMS *m*/*z* 180 [M + H]^+^.

Staurosporine (**12**): Amorphous powder, ^1^H (400 MHz, CDCl_3_): 9.41 (1H, d, *J* = 7.6 Hz, H-4), 7.92 (1H, d, *J* = 8.4 Hz, H-11), 7.88 (1H, d, *J* = 7.6 Hz, H-8), 7.48 (1H, t, *J* = 8.0 Hz, H-2), 7.42 (1H, t, *J* = 8.0 Hz, H-10), 7.36 (1H, t, *J* = 8.0 Hz, H-3), 7.31 (1H, t, *J* = 8.0 Hz, H-9), 7.28 (1H, t, *J* = 8.0 Hz, H-1), 6.55 (1H, d, *J* = 5.2 Hz, H-6**′**), 5.01 (2H, s, H-7), 3.87 (1H, d, *J* = 3.2 Hz, H-3**′**), 3.40 (3H, s, H-3**′**-OMe), 3.34 (1H, q, *J* = 3.6 Hz, H-4**′**), 2.74 (1H, dd, *J* = 14.8, 3.2 Hz, H-5**′**), 2.39 (1H, ddd, *J* = 14.4, 6.0, 3.6 Hz, H-5**′**), 2.35 (3H, s, H-2**′**-OMe), 1.55 (3H, s, H-4**′**-NMe).

### 3.4. MTT Proliferation Assay

MTT assay was used to assess the proliferation of Molt-4 (human T lymphoblast, acute lymphoblastic leukemia) cells after treatment with all fractions and compounds. The Molt 4 cell lines were obtained from the American Type Culture Collection (ATCC, Manassas, VA, USA). Culture plates with 96 wells were used for the MTT assay. For the anti-proliferative assessments in Figure 1, Molt-4 cells were seeded at 2 × 10^5^ per well and were treated with 1 μg/mL of all fractions. For the evaluations in Table 2, Molt-4 cells were seeded at 2 × 10^5^ per well and were treated with 100, 50, 25, and 12.5 μM of the tested DKPs (1–11); with 10, 1, 0.1, 0.01, and 0.001 μM of the compound 12 and positive control (Doxorubicin).The anti-proliferative effect of the test fractions and compounds was determined by MTT cell proliferation assay (thiazolyl blue tetrazolium bromide, Sigma-M2128) for 24, 48, or 72 h. An ELISA reader (Anthoslabtec Instrument, Salzburg, Austria) was used to measure light absorbance values (OD = OD_570_ − OD_620_) at 570 and 620 nm. Calculations were performed to determine the concentration that led to 50% inhibition (IC_50_). The results were expressed as a percentage of the control ± SD established from *n* = 3 wells per experiment, from three independent experiments.

### 3.5. ChemGPS-NP Analysis

The chemical global positioning system is a public tool for navigation in biologically relevant chemical space. It has eight principal components (PCs) based on 35 carefully selected chemical descriptors describing physical–chemical properties, including size, shape, polarizability, lipophilicity, polarity, and hydrogen bond capacity. The ChemGPS-NP score predictions were calculated through an online tool, ChemGPS-NPWeb (http://chemgps.bmc.uu.se (accessed on 2 November 2021)) based on their structural information as a simplified molecular input line entry specification (SMILES), derived via ChemBioDraw version 16.0 (CambridgeSoft, Waltham, MA, USA). The involvements of chemotherapeutic agents were categorized according to the ChEMBL database using keywords of alkylating agents, anti-metabolites, proteasome inhibitors, tyrosine kinase inhibitors, topoisomerase, and anti-microtubule agents, and were plotted into the ChemGPS-NP chemical space using the software Grapher 2.6 (Mac OS, Cupertino, CA, USA).

## 4. Conclusions

We identified anti-leukemic metabolites from marine-derived *Streptomyces* sp. LY1209 and determined their possible molecular targets. A series of diketopiperazines (DKPs) were identified including one novel (**1**) and two known compounds (**9** and **10**) isolated for the first time from natural resources. An indolocarbazole derivative exhibited the most potent anti-proliferative activity. A chemo-informatic approach, ChemGPS-NP analysis, illustrated the possible molecular targets of these bioactive metabolites. The strategy developed in this study can be employed to facilitate the future discovery of potential anti-leukemic drug leads.

## Figures and Tables

**Table 1:** ^1^H (500 MHz, CD_3_OD) and ^13^C (125 MHz, CD_3_OD) NMR data for **1**.

Position	*δ*_H_ (*J* in Hz) ^a^	*δ*_C_ (Mult.) ^b^	^1^H–^1^H COSY	HMBC
2		167.0 (C)		
3	4.23, td (3.9, 1.3) ^c^	57.2 (CH) ^d^	H-11	C-2, C-5
5		169.9 (C)		
6	2.54, dd (11.3, 1.6)	58.4 (CH)	H-7	
7	1.32, m; 2.09, d (11.0)	31.6 (CH_2_)		
8	1.33, m; 1.83, m	24.8 (CH_2_)	H-7, H-9	
9	1.33, m; 1.65, m	25.3 (CH_2_)	H-7, H-8, H-10	
10	2.20, td (12.9, 3.2);	43.3 (CH_2_)	H-8, H-9	
	4.50, dt (13.5, 2.0)			
11	3.17, dd (14, 1.8)	40.9 (CH_2_)	H-3	C-2, C-3, C-12, C-13
	4.50, dt (13.5, 2.0)			
12		126.5 (C)		
13, 13‘	6.91, d (8.5)u	132.5 (CH)	H-14	C-11, C-15
14, 14‘	6.70, d (8.5)	116.2 (CH)	H-13	C-12, C-15
15		158.2 (C)		

^a^ 500 MHz in CDCl_3_; ^b^ 125 MHz in CDCl_3_; ^c^ *J* values (Hz) are given in parentheses; ^d^ Numbers of the attached protons were deduced by DEPT experiments.

**Table 2:** Anti-proliferative activity of the isolated metabolites from the ethyl EtOAc extract of *Streptomyces* sp. LY1209.

Compounds	Cell Lines (IC_50_ μM)
	Molt 4
**1**	95.93 ± 2.32
**2**	– ^a^
**3**	– ^a^
**4**	– ^a^
**5**	– ^a^
**6**	56.23 ± 7.52
**7**	– ^a^
**8**	– ^a^
**9**	– ^a^
**10**	– ^a^
**11**	– ^a^
**12**	0.01 ± 0.001
Doxorubicin ^b^	0.04

^a^ IC_50_ > 100 μM for 72 h; ^b^ positive control.

## Data Availability

The data presented in this study are available in the article and Supplementary Materials.

## Supplementary Materials

The following supporting information can be downloaded at: https://www.mdpi.com/article/10.3390/metabo12040320/s1, Table S1: 1D and 2D NMR (600 MHz, CD3OD) data of **1**, Table S2: 1D NMR (500 MHz, CD3OD) data of **2**, Table S3: 1D NMR (500 MHz, CD3OD) data of **3**, Table S4: 1D (500 MHz, CD3OD) data of **4**, Table S5: 1D NMR (500 MHz, CD3OD) data of **5**, Table S6: 1D (500 MHz, CD3OD) data of **6**, Table S7: 1D NMR (500 MHz, CD3OD) data of **7**, Table S8: 1D NMR (500 MHz, CD3OD) data of **8**, Table S9: 1D NMR (500 MHz, CD3OD) data of **9**, Table S10: 1D NMR (500 MHz, CD3OD) data of **10**, Table S11: 1D NMR (500 MHz, CD3OD) data of **11**; Figure S1: IR spectrum of **1**, Figure S2: UV-VIS spectrum of **1**, Figure S3: HREMS spectrum of **1**, Figure S4: ^1^H NMR (500 MHz, CD_3_OD) spectrum of **1**, Figure S5: ^1^H NMR (500 MHz, CD_3_OD) spectrum of **1** (Partial enlarged view), Figure S6: ^13^C NMR (125 MHz, CD_3_OD) spectrum of **1**, Figure S7: DEPT spectrum of **1**, Figure S8: HSQC spectrum of **1**, Figure S9: HMBC spectrum of **1**, Figure S10: COSY spectrum of **1**, Figure S11: NOESY spectrum of **1**, Figure S12: ESIMS spectrum of **2**, Figure S13: ^1^H NMR (500 MHz, CD_3_OD) spectrum of **2**, Figure S14: ^13^C NMR (125 MHz, CD_3_OD) spectrum of **2**, Figure S15: DEPT spectrum of **2**, Figure S16: ESIMS spectrum of **3**, Figure S17: ^1^H NMR (500 MHz, CD_3_OD) spectrum of **3**, Figure S18: ESIMS spectrum of **4**, Figure S19: ^1^H NMR (500 MHz, CD_3_OD) spectrum of **4**, Figure S20: ^13^C NMR (125 MHz, CD_3_OD) spectrum of **4**, Figure S21: DEPT spectrum of **4**, Figure S22: ESIMS spectrum of **5**, Figure S23: ^1^H NMR (500 MHz, CD_3_OD) spectrum of **5**, Figure S24: ^13^C NMR (125 MHz, CD_3_OD) spectrum of **5**, Figure S25: DEPT spectrum of **5**, Figure S26: ESIMS spectrum of **6**, Figure S27: ^1^H NMR (500 MHz, CD_3_OD) spectrum of **6**, Figure S28: ^13^C NMR (125 MHz, CD_3_OD) spectrum of **6**, Figure S29: DEPT spectrum of **6**, Figure S30: ESIMS spectrum of **7**, Figure S31: ^1^H NMR (500 MHz, CD_3_OD) spectrum of **7**, Figure S32: ^13^C NMR (125 MHz, CD_3_OD) spectrum of **7**, Figure S33: DEPT spectrum of **7**, Figure S34: ESIMS spectrum of **8**, Figure S35: ^1^H NMR (500 MHz, CD_3_OD) spectrum of **8**, Figure S36: ^13^C NMR (125 MHz, CD_3_OD) spectrum of **8**, Figure S37: DEPT spectrum of **8**, Figure S38: ESIMS spectrum of **9**, Figure S39: ^1^H NMR (500 MHz, CD_3_OD) spectrum of **9**, Figure S40: ^13^C NMR (125 MHz, CD_3_OD) spectrum of **9**, Figure S41: DEPT spectrum of **9**, Figure S42: ^1^H NMR (500 MHz, CD_3_OD) spectrum of **10**, Figure S43: ^13^C NMR (125 MHz, CD_3_OD) spectrum of **10**, Figure S44: DEPT spectrum of **10**, Figure S45: ESIMS spectrum of **11**, Figure S46: ^1^H NMR (500 MHz, CD_3_OD) spectrum of **11**, Figure S47: ^13^C NMR (125 MHz, CD_3_OD) spectrum of **11**, Figure S48: DEPT spectrum of **11**.
