# Supplementary material for: Probing Anti-Leukemic Metabolites from Marine-Derived Streptomyces sp. LY1209
Source: Metabolites. 2022 Apr 2;12(4):320. doi: 10.3390/metabo12040320 (PMC9025307; doi:10.3390/metabo12040320)
Supplement: Supplementary file 1 [file metabolites-12-00320-s001.zip › metabolites-1657759 (Supplementary revised).pdf]

SUPPORTING INFORMATION

Article

Probing Anti-Leukemic Metabolites from Marine-Derived *Streptomyces* sp. LY1209

You-Ying Chen [1,†], Lo-Yun Chen [2,†], Po-Jen Chen [3,†],Mohamed El-Shazly [4,5], Bo-Rong Peng [6], Yu-Cheng Chen [7], Chun-Han Su [8], Jui-Hsin Su [1,6], Ping-Jyun Sung [1,6,9], Pei-Tzu Yen [10], Lung-Shuo Wang [10,11,*] and Kuei-Hung Lai [2,12,13,*]

Table of Contents

Figure S1. IR spectrum of **1**.

Figure S2. UV-VIS spectrum of **1**.

Figure S3. HREMS spectrum of **1**.

Figure S4. ^{1}H NMR (500 MHz, CD_3OD) spectrum of **1**.

Figure S5. ^{1}H NMR (500 MHz, CD_3OD) spectrum of **1** (Partial enlarged view) .

Figure S6. ^{13}C NMR (125 MHz, CD_3OD) spectrum of **1**.

Figure S7. DEPT spectrum of **1**.

Figure S8. HSQC spectrum of **1**.

Figure S9. HMBC spectrum of **1**.

Figure S10. COSY spectrum of **1**.

Figure S11. NOESY spectrum of **1**.

Figure S12. ESIMS spectrum of **2**.

Figure S13. ^{1}H NMR (500 MHz, CD_3OD) spectrum of **2**.

Figure S14. ^{13}C NMR (125 MHz, CD_3OD) spectrum of **2**.

Figure S15. DEPT spectrum of **2**.

Figure S16. ESIMS spectrum of **3**.

Figure S17. ^{1}H NMR (500 MHz, CD_3OD) spectrum of **3**.

Figure S18. ESIMS spectrum of **4**.

Figure S19. ^{1}H NMR (500 MHz, CD_3OD) spectrum of **4**.

Figure S20. ^{13}C NMR (125 MHz, CD_3OD) spectrum of **4**.

Figure S21. DEPT spectrum of **4**.

Figure S22. ESIMS spectrum of **5**.

Figure S23. ^{1}H NMR (500 MHz, CD_3OD) spectrum of **5**.

Figure S24. ^{13}C NMR (125 MHz, CD_3OD) spectrum of **5**.

Figure S25. DEPT spectrum of **5**.

Figure S26. ESIMS spectrum of **6**.

Figure S27. ^{1}H NMR (500 MHz, CD_3OD) spectrum of **6**.

Figure S28. ^{13}C NMR (125 MHz, CD_3OD) spectrum of **6**.

Figure S29. DEPT spectrum of **6**.

Figure S30. ESIMS spectrum of **7**.

Figure S31. ^{1}H NMR (500 MHz, CD_3OD) spectrum of **7**.

Figure S32. ^{13}C NMR (125 MHz, CD_3OD) spectrum of **7**.

Figure S33. DEPT spectrum of **7**.

Figure S34. ESIMS spectrum of **8**.

Figure S35. ^{1}H NMR (500 MHz, CD_3OD) spectrum of **8**.

Figure S36. ^{13}C NMR (125 MHz, CD_3OD) spectrum of **8**.

Figure S37. DEPT spectrum of **8**.

Figure S38. ESIMS spectrum of **9**.

Figure S39. ^{1}H NMR (500 MHz, CD_3OD) spectrum of **9**.

Figure S40. ^{13}C NMR (125 MHz, CD_3OD) spectrum of **9**.

Figure S41. DEPT spectrum of **9**.

Figure S42. ^{1}H NMR (500 MHz, CD_3OD) spectrum of **10**.

Figure S43. ^{13}C NMR (125 MHz, CD_3OD) spectrum of **10**.

Figure S44. DEPT spectrum of **10**.

Figure S45. ESIMS spectrum of **11**.

Figure S46. ^{1}H NMR (500 MHz, CD_3OD) spectrum of **11**.

Figure S47. ^{13}C NMR (125 MHz, CD_3OD) spectrum of **11**.

Figure S48. DEPT spectrum of **11**.

Table S1. 1D and 2D NMR (600 MHz, CD_3OD) data of **1**.

Table S2. 1D NMR (500 MHz, CD_3OD) data of **2**.

Table S3. 1D NMR (500 MHz, CD_3OD) data of **3**.

Table S4. 1D NMR (500 MHz, CD_3OD) data of **4**.

Table S5. 1D NMR (500 MHz, CD_3OD) data of **5**.

Table S6. 1D NMR (500 MHz, CD_3OD) data of **6**.

Table S7. 1D NMR (500 MHz, CD_3OD) data of **7**.

Table S8. 1D NMR (500 MHz, CD_3OD) data of **8**.

Table S9. 1D NMR (500 MHz, CD_3OD) data of **9**.

Table S10. 1D NMR (500 MHz, CD_3OD) data of **10**.

Table S11. 1D NMR (500 MHz, CD_3OD) data of **11**.

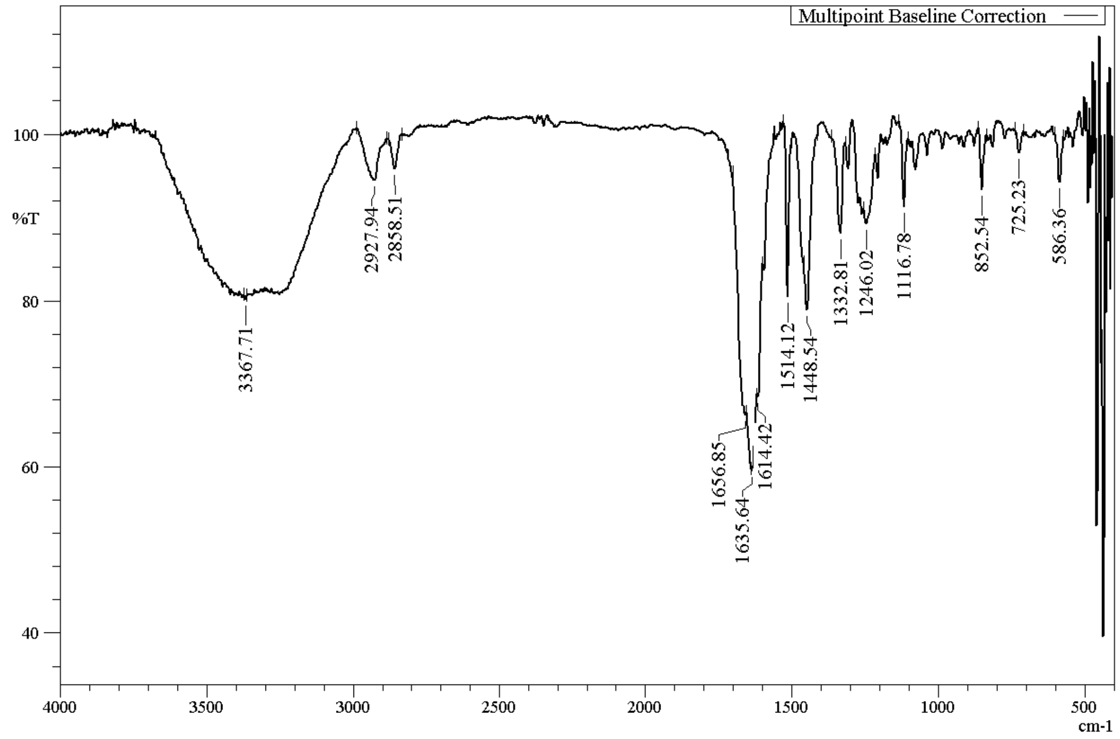

Figure S1. IR spectrum of **1**.

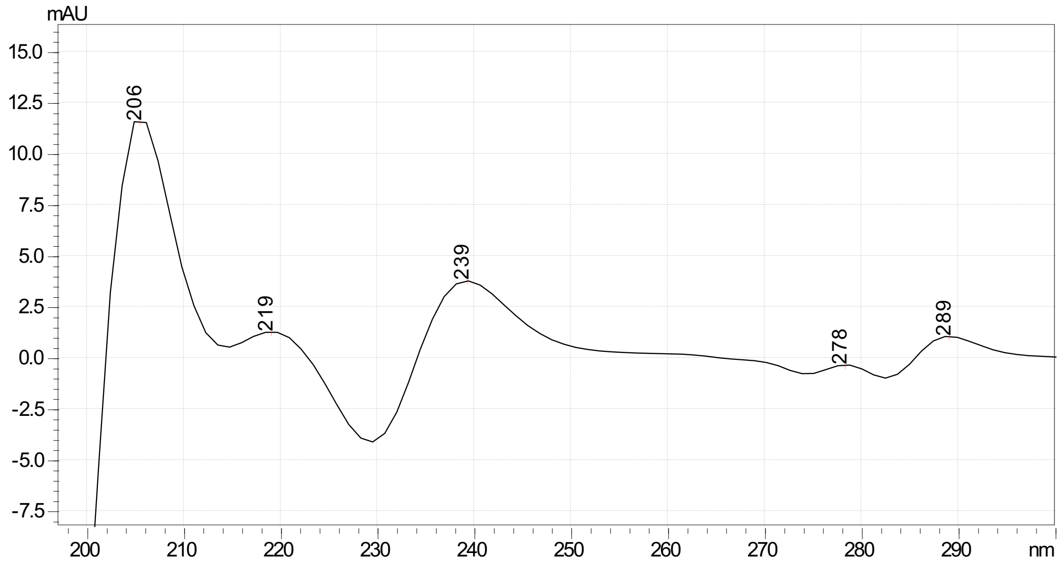

Figure S2. UV-VIS spectrum of **1**.

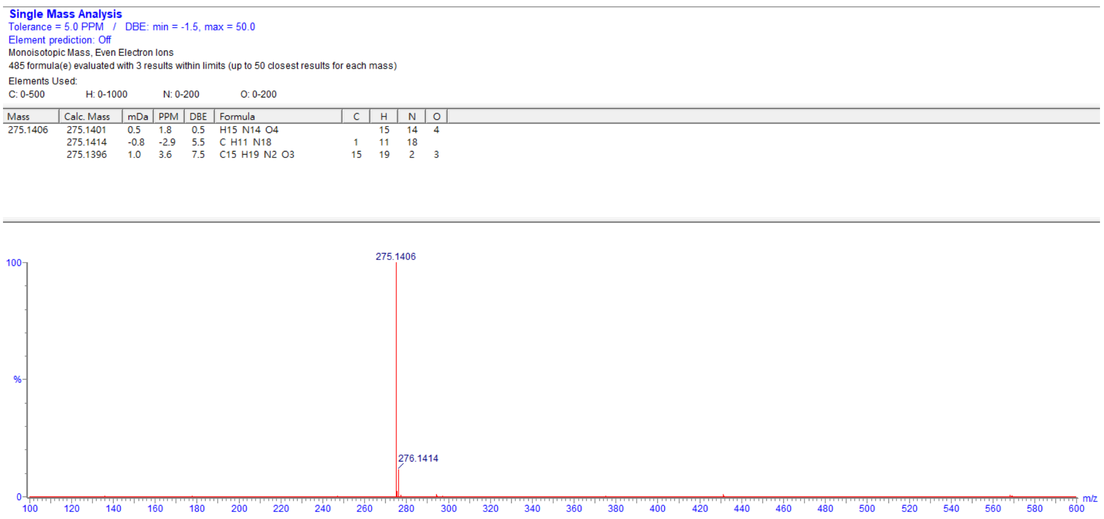

Figure S3. HREMS spectrum of **1**.

Table S1. 1D and 2D NMR (600 MHz, CD_3OD) data of **1**.

**Pos.**	**δ_H, mult (*J* in Hz)**	δ_C (Mult.) [b]	^1H–^1H COSY	HMBC
1				
2		167.0 (C)		
3	4.23, td (3.9, 1.3)	57.2 (CH)	H-11	C-2, C-5
4				
5		169.9 (C)		
6	2.54, dd (11.3, 1.6)	58.4 (CH)	H-7	
7	1.32, m; 2.09, d (11.0)	31.6 (CH_2)		
8	1.33, m; 1.83, m	24.8 (CH_2)	H-7, H-9	
9	1.33,m; 1.65, m	25.3 (CH_2)	H-7, H-8, H-10	
10	2.20, td (12.9, 3.2);	43.3 (CH_2)	H-8, H-9	
	4.50, dt (13.5, 2.0)			
11	3.17, dd (14, 1.8)	40.9 (CH_2)	H-3	C-2, C-3, C-12, C13
	4.50, dt (13.5, 2.0)			
12		126.5 (C)		
13, 13′	6.91, d (8.5)	132.5 (CH)	H-14	C-11, C-15
14, 14′	6.70, d (8.5)	116.2 (CH)	H-13	C-12, C-15
15		158.2 (C)		

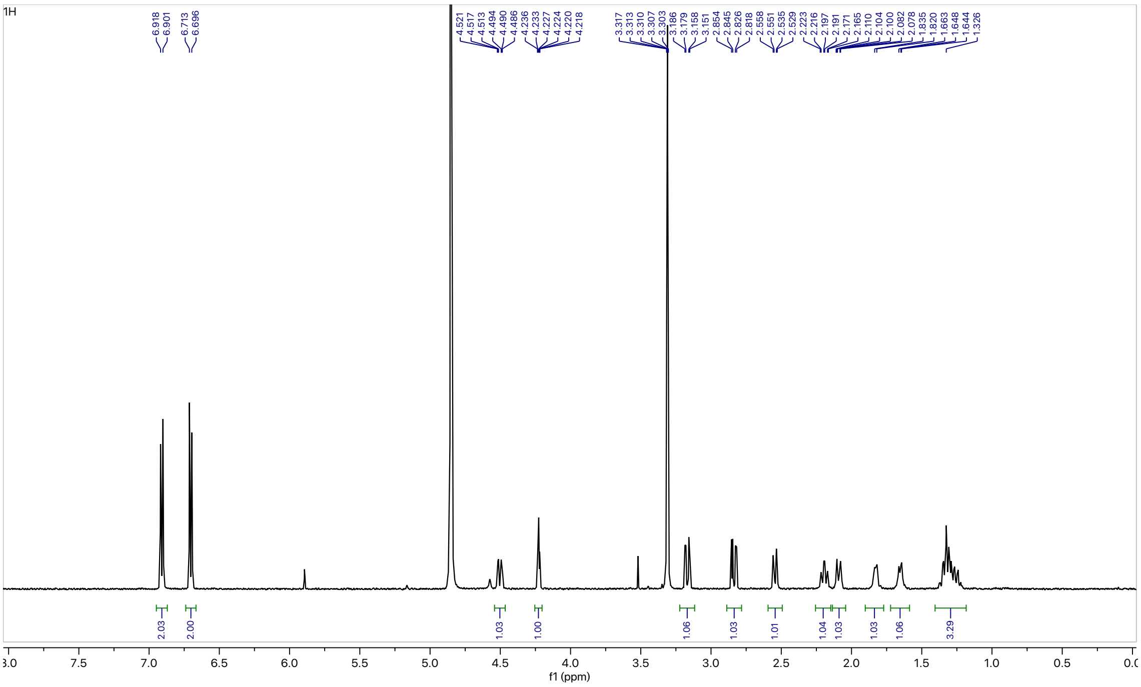

Figure S4. ^1H NMR (500 MHz, CD_3OD) spectrum of **1**.

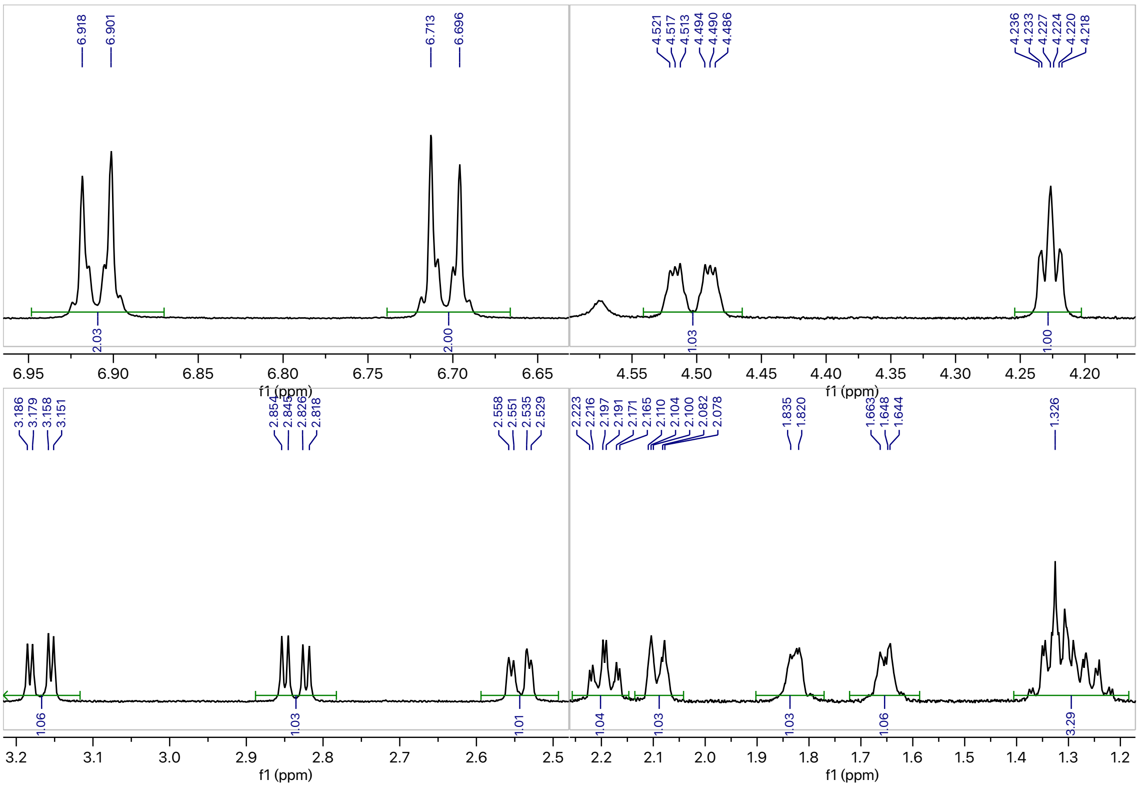

Figure S5. ^1H NMR (500 MHz, CD_3OD) spectrum of **1** (Partial enlarged view) .

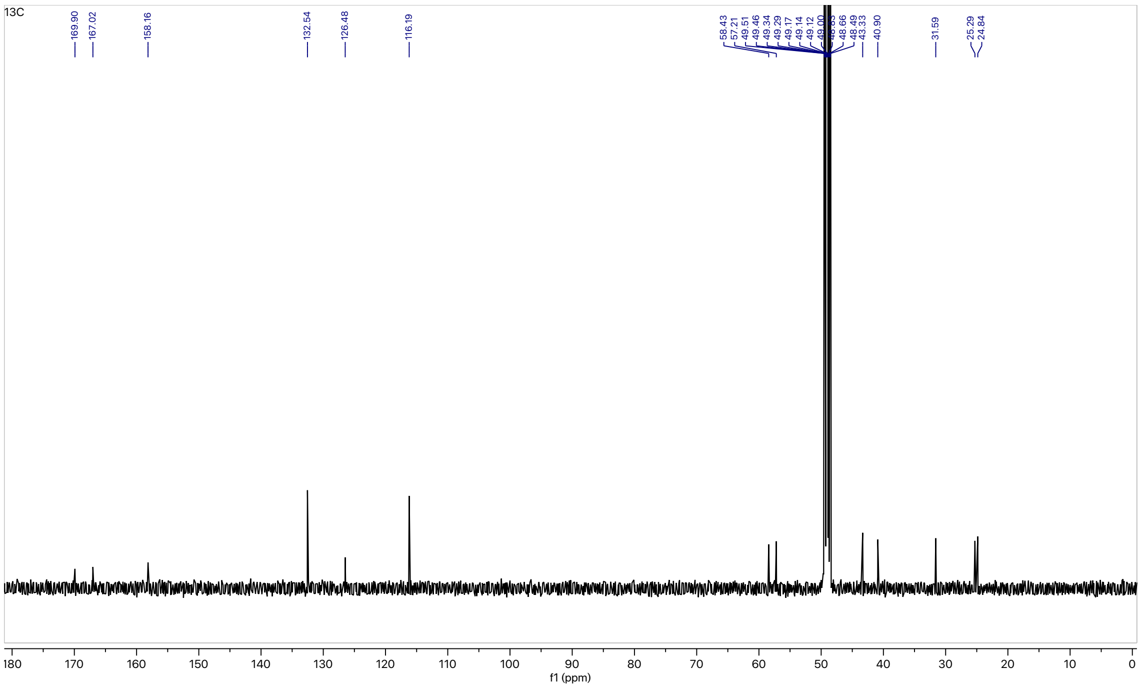

Figure S6. ^{13}C NMR (125 MHz, CD_3OD) spectrum of **1**.

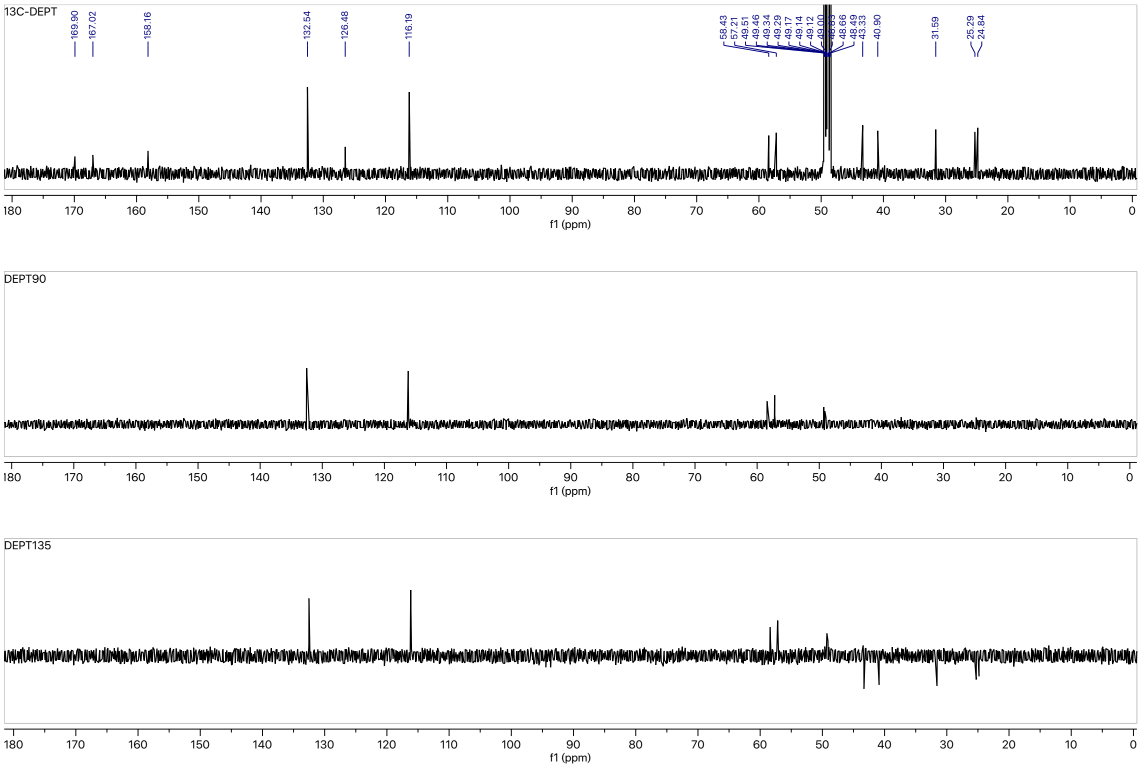

Figure S7. DEPT spectrum of **1**.

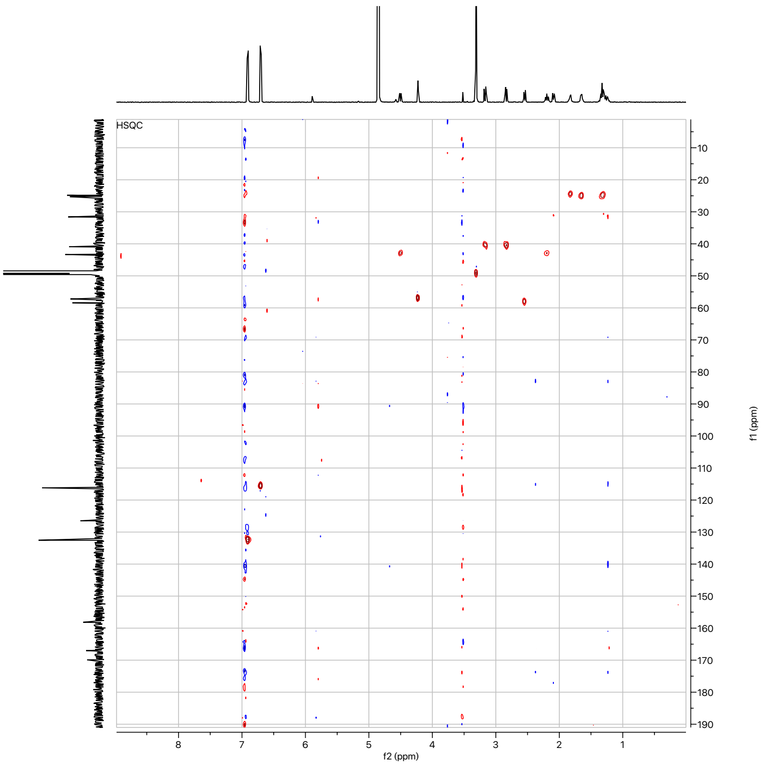

Figure S8. HSQC spectrum of **1**.

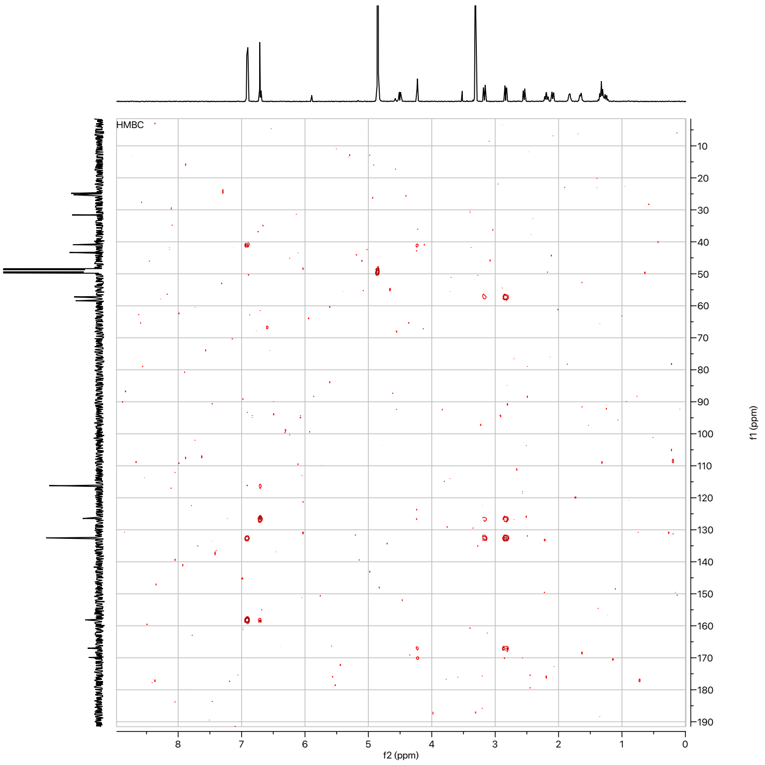

Figure S9. HMBC spectrum of **1**.

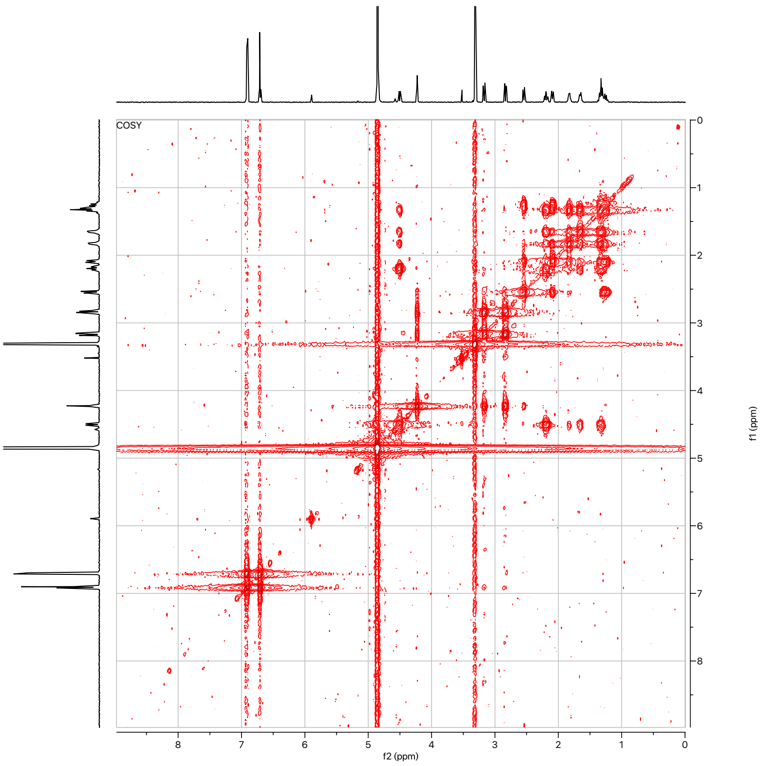

Figure S10. COSY spectrum of **1**.

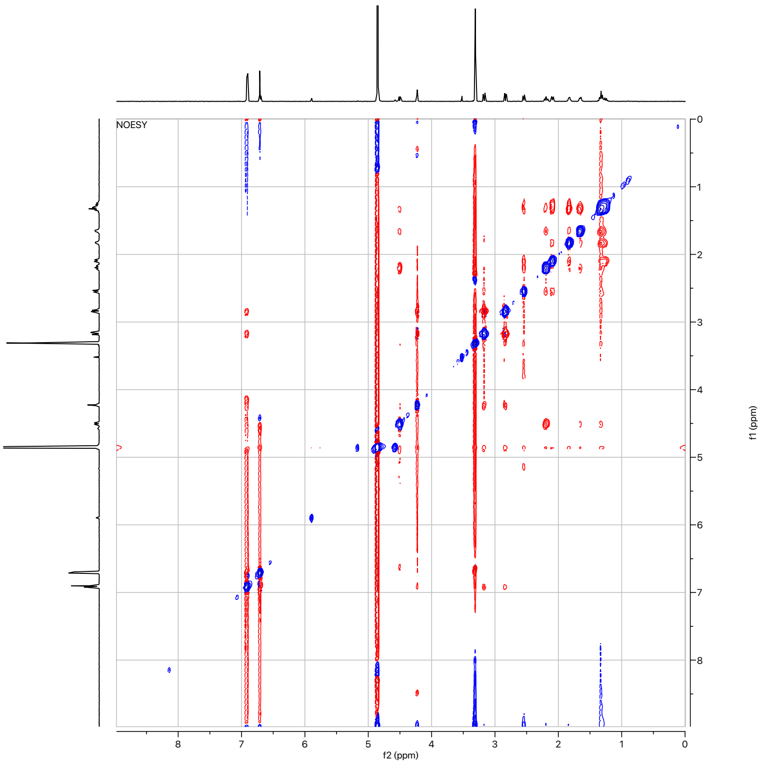

Figure S11. NOESY spectrum of **1**.

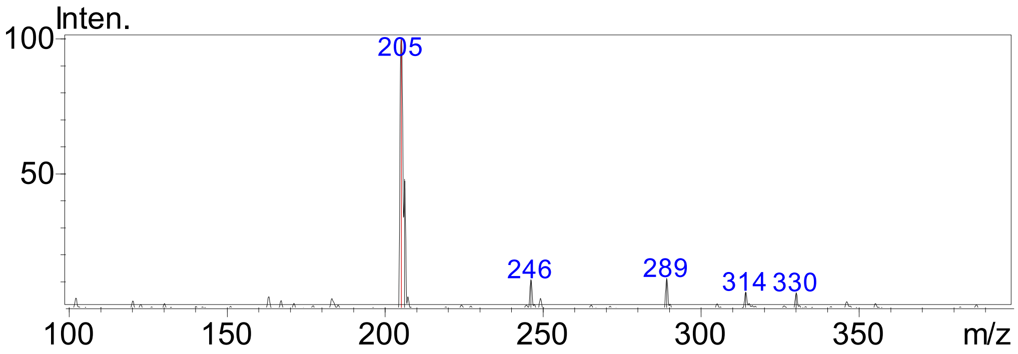

Figure S12. ESIMS spectrum of **2**.

Table S2. 1D NMR (500 MHz, CD_3OD) data of **2**.

**Pos.**	δ_H, mult (*J* in Hz)	δ_C (Mult.)
1		
2		168.8(C)
3	4.23, t (4.5)	57.7 (CH)
4		
5		170.2 (C)
6	2.65, dd (17.5, 0.5)	44.8 (CH_2)
	3.43, dd (17.5, 0.5)	
7	3.00, dd (13.8, 2.4)	41.0 (CH_2)
	3.24, dd (14.0, 2.0)	
8		136.5 (C)
9, 9′	7.22, dd (6.8, 1.4)	131.6 (CH)
10, 10′	7.30, m	129.8 (CH)
11	7.31, m	128.6 (CH)

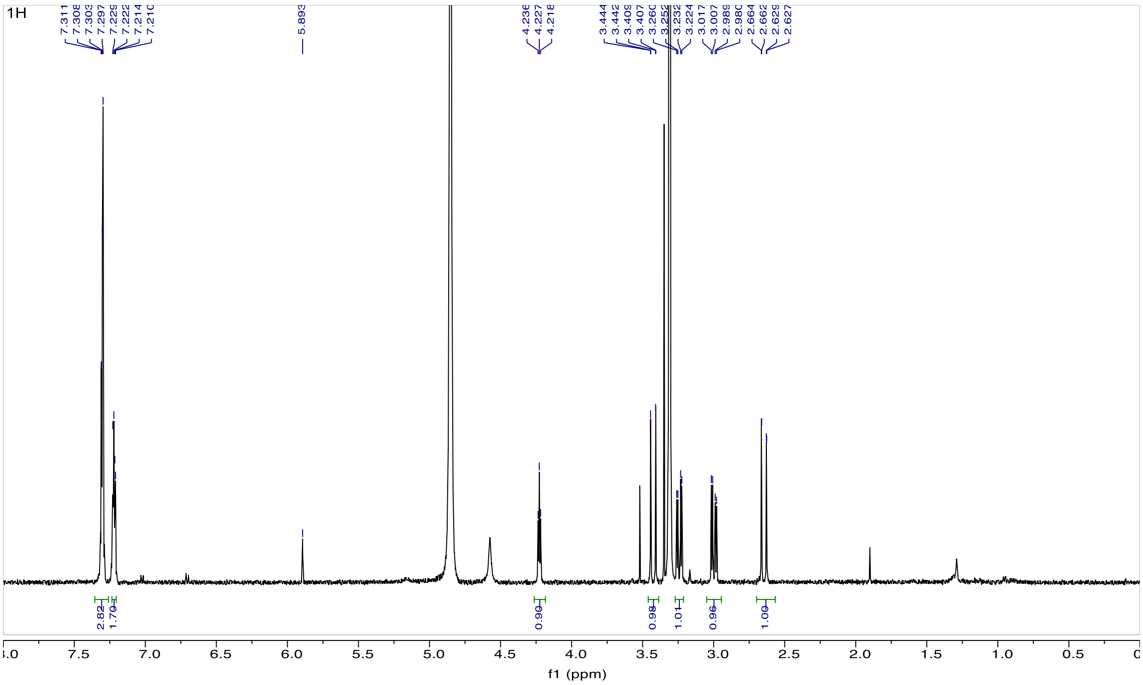

Figure S13. ^{1}H NMR (500 MHz, CD_3OD) spectrum of **2**.

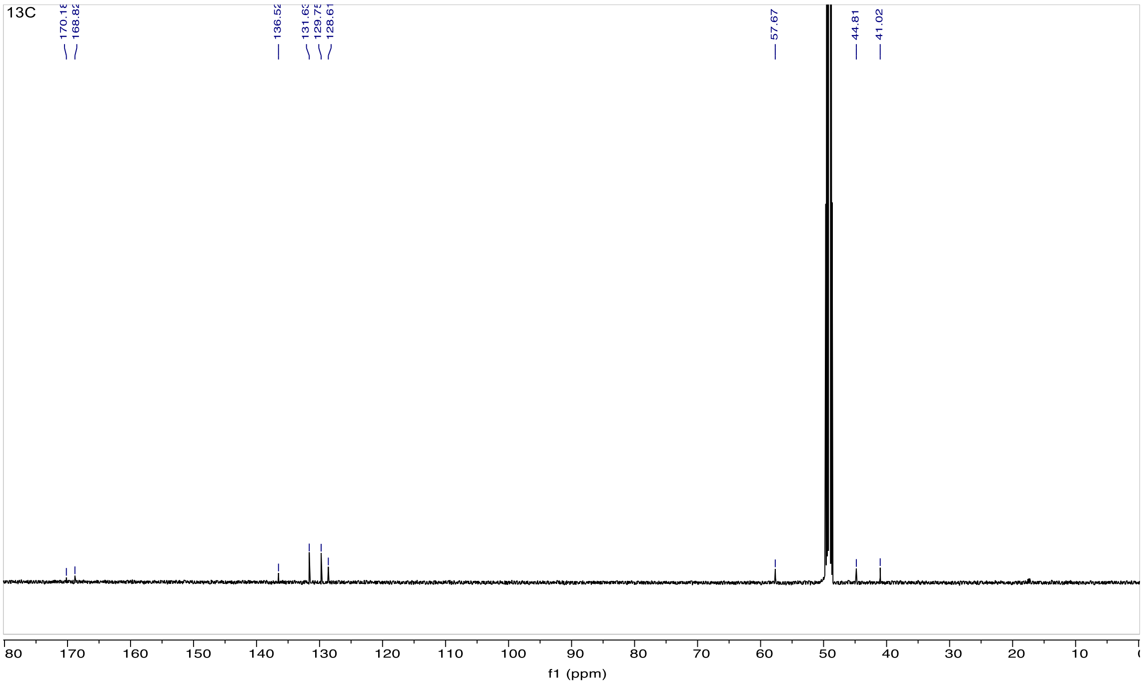

Figure S14. ^{13}C NMR (125 MHz, CD_3OD) spectrum of **2**.

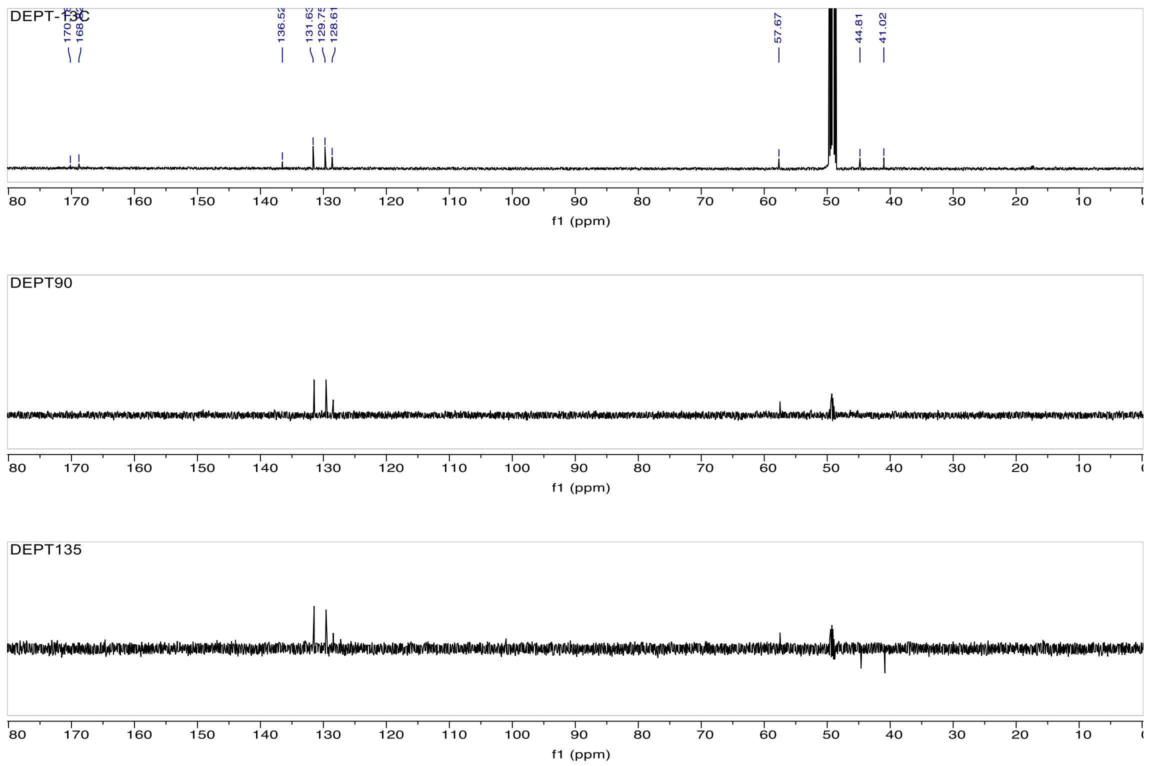

Figure S15. DEPT spectrum of **2**.

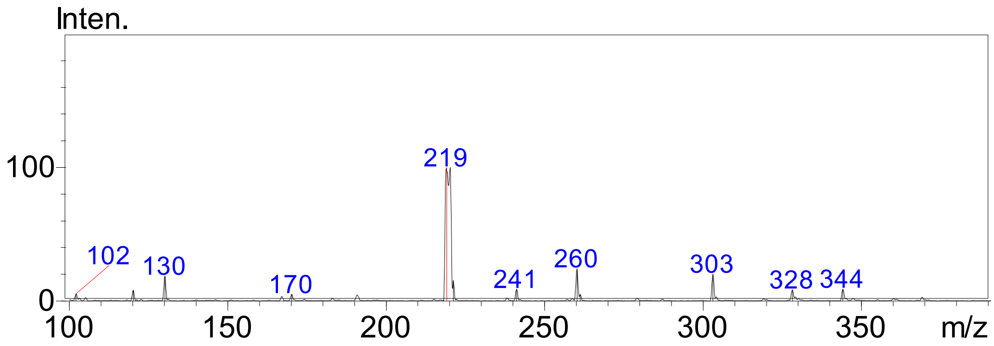

Figure S16. ESIMS spectrum of **3**.

Table S3. 1D NMR (500 MHz, CD_3OD) data of **3**.

Pos.	δ_H, mult (*J* in Hz)
1	
2	
3	4.31, ddd (6.0, 4.5, 1.5)
4	
5	
6	3.76, ddd (10.5, 9.0, 2.0)
7	2.96, dd (17.0, 6.0); 3.28, m
8	
9, 9′	
10, 10′	7.20-7.32, m
11	
12	0.52, d (9.0)

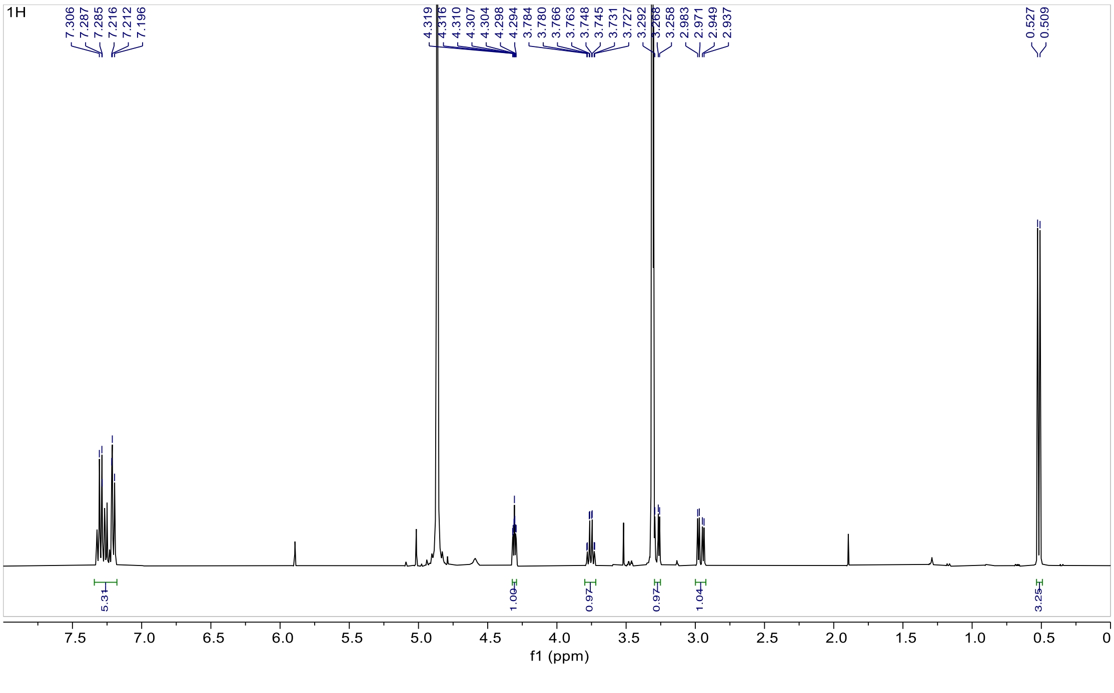

Figure S17. ^1H NMR (500 MHz, CD_3OD) spectrum of **3**.

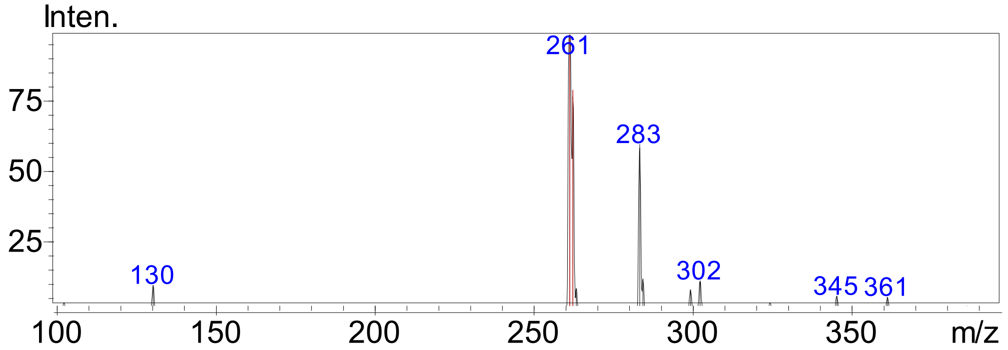

Figure S18. ESIMS spectrum of **4**.

Table S4. 1D (500 MHz, CD_3OD) data of **4**.

**Pos.**	δ_H, mult (*J* in Hz)	δ_C (Mult.)
1		
2		167.0 (C)
3	4.36, td (4.8, 2.0)	57.9 (CH)
4		
5		170.8 (C)
6	4.05, ddd (10.8, 6.5, 2.0)	60.1 (CH)
7	3.06, ddd (19.5, 14.5, 5.5)	37.7 (CH_2)
8		127.6 (C)
9, 9′	7.04, d (8.5)	132.2 (CH)
10, 10′	6.70, d (8.5)	116.3 (CH)
11		157.7 (C)
14	1.23, m; 2.10, m	29.4 (CH_2)
15	1.80, m	22.7 (CH_2)
16	3.36, m; 3.55, m	45.9 (CH_2)

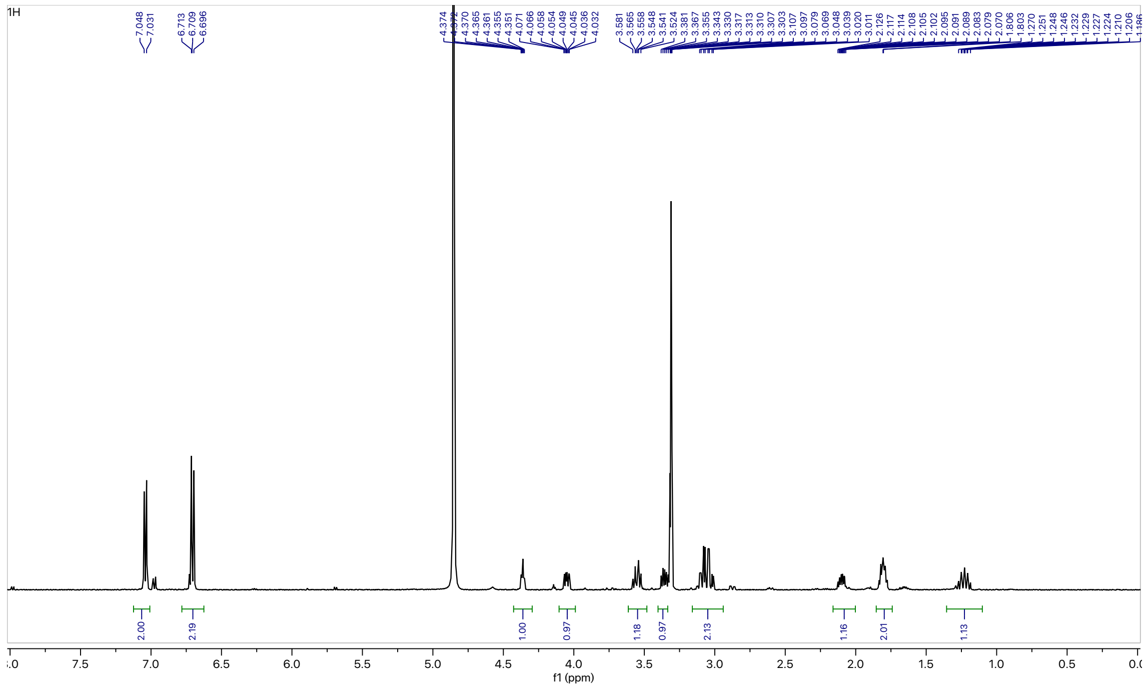

Figure S19. ^{1}H NMR (500 MHz, CD_3OD) spectrum of **4**.

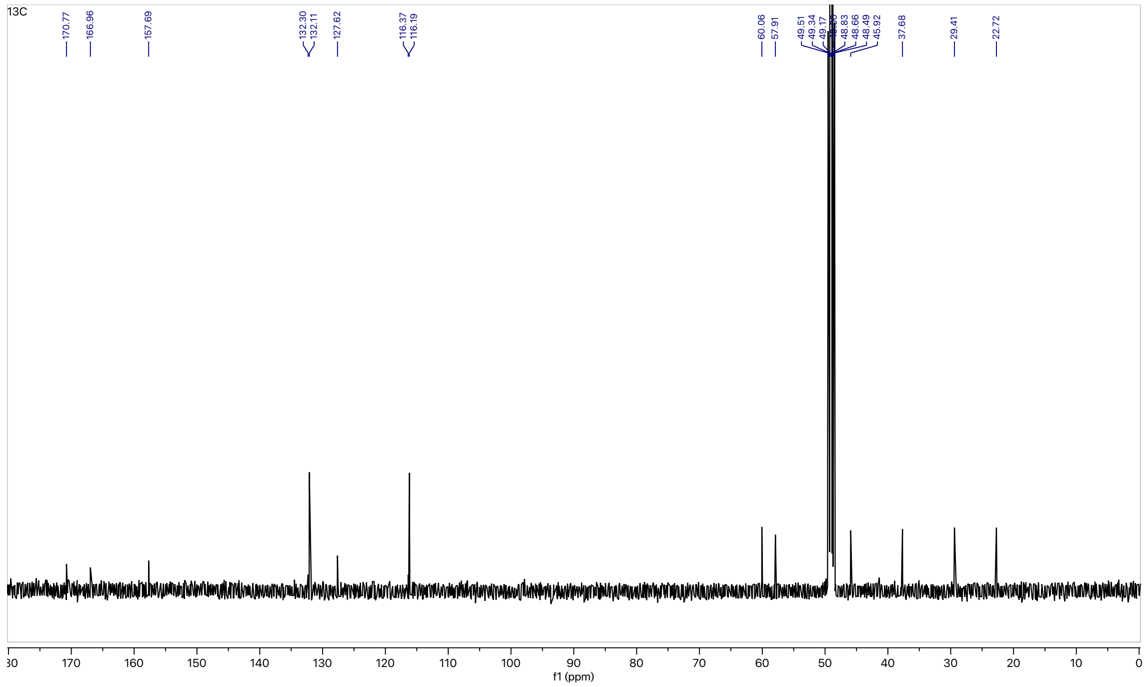

Figure S20. ^{13}C NMR (125 MHz, CD_3OD) spectrum of **4**.

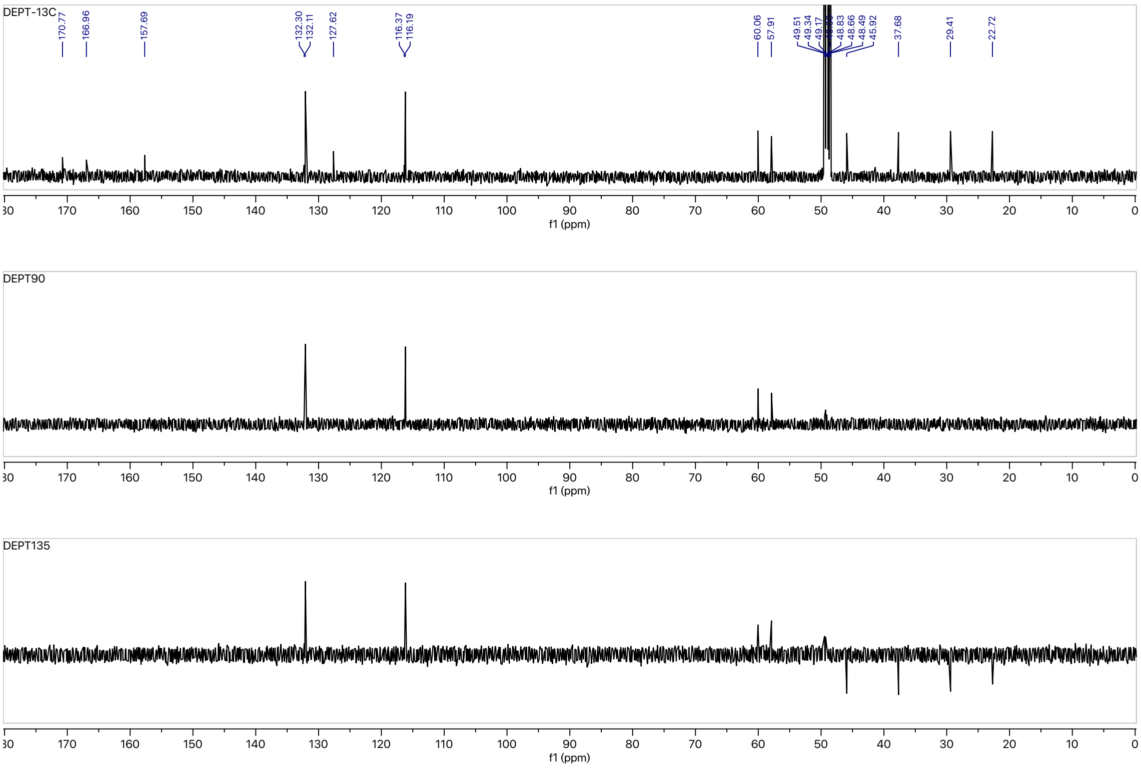

Figure S21. DEPT spectrum of **4**.

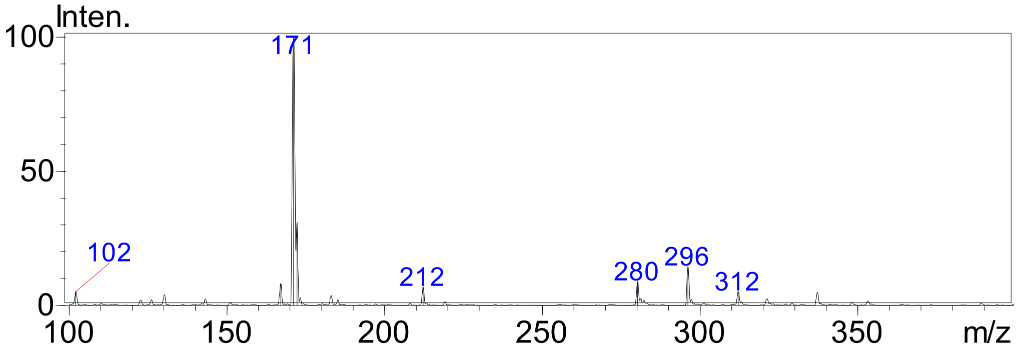

Figure S22. ESIMS spectrum of **5**.

Table S5. 1D NMR (500 MHz, CD_3OD) data of **5**.

**Pos.**	δ_H, mult (J in Hz)	δ_C (Mult.)
1		
2		171.5 (C)
3	3.84 dd (3.7, 1.2)	61.6 (CH)
4		
5		171.1 (C)
6	4.05 ddd (14.0, 6.7, 1.2)	51.9 (CH)
7	2.29 m	33.5 (CH)
8	1.07 d (6.7)	21.3 (CH_3)
9	0.97 d (6.7)	19.2 (CH_3)
10	1.45 d (7.0)	17.4 (CH_3)

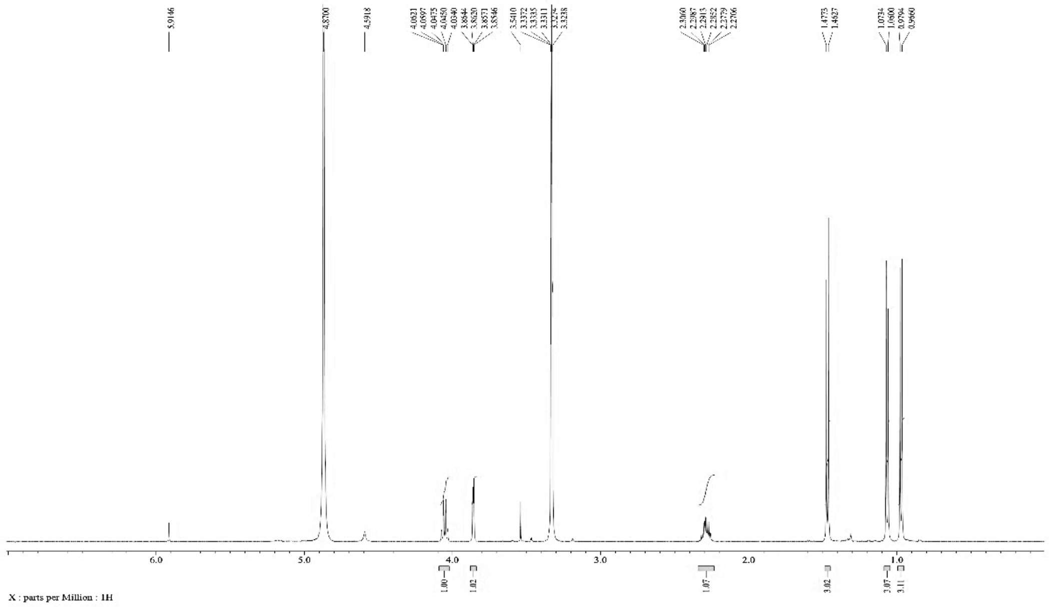

Figure S23. ^{1}H NMR (500 MHz, CD_3OD) spectrum of **5**.

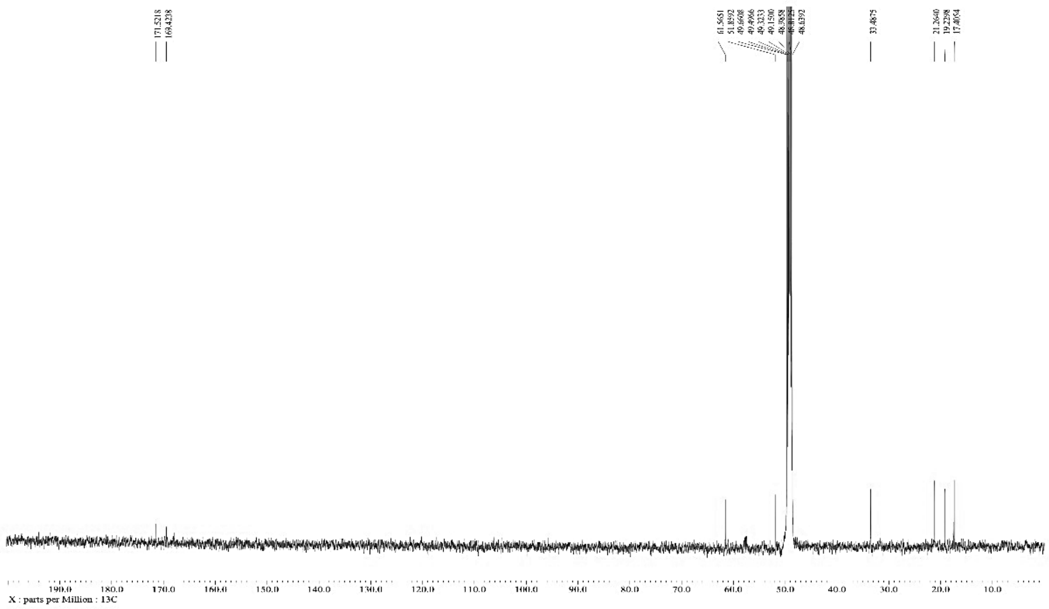

Figure S24. ^{13}C NMR (125 MHz, CD_3OD) spectrum of **5**.

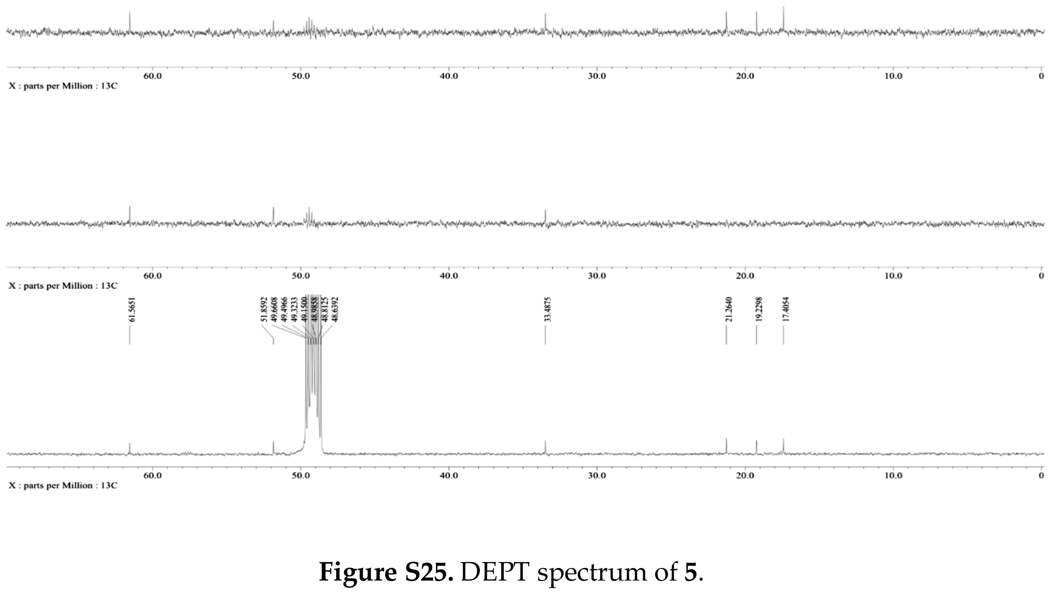

Figure S25. DEPT spectrum of **5**.

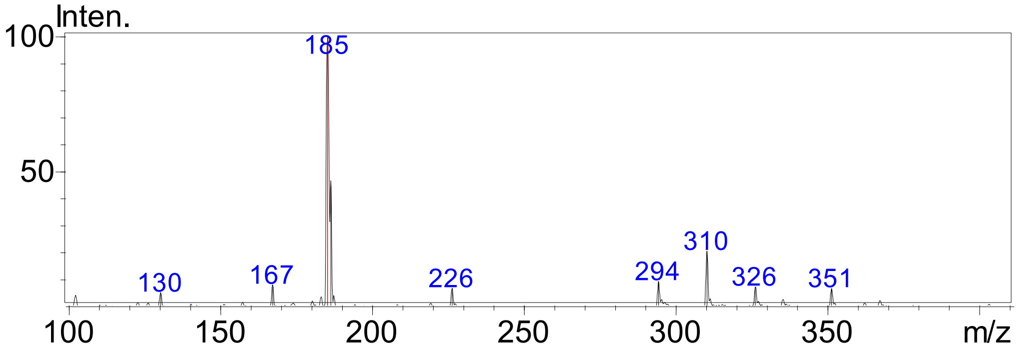

Figure S26. ESIMS spectrum of **6**.

Table S6. 1D (500 MHz, CD_3OD) data of **6**.

**Pos.**	δ_H, mult (*J* in Hz) [a]	δ_C (Mult.) [b]
1		
2		169.4 (C)
3	3.91 dd (3.5, 0.8)	52.1 (CH)
4		
5		171.4 (C)
6	4.03 ddd (14.0, 7.0, 1.5)	51.8 (CH)
7	1.96, m	40.4 (CH)
8	1.26, m; 1.52, m	25.8 (CH_2)
9	0.95, t (7.3)	12.3(CH_3)
10	1.03, d (7.0)	15.7 (CH_3)
11	1.45, d (7.0)	21.1 (CH_3)

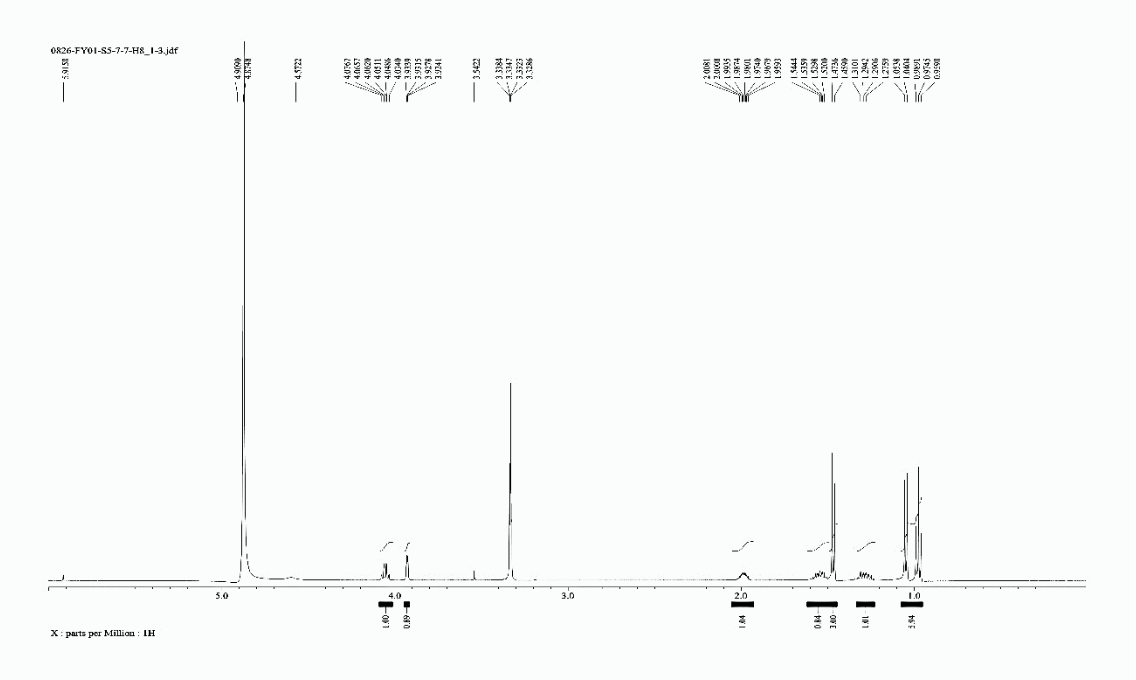

Figure S27. ^{1}H NMR (500 MHz, CD_3OD) spectrum of **6**.

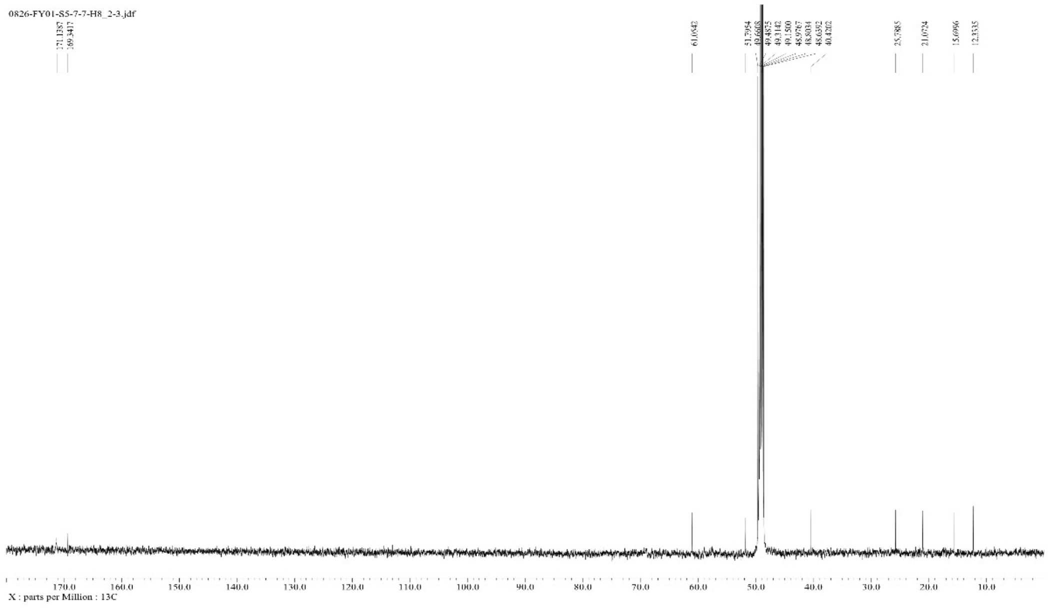

Figure S28. ^{13}C NMR (125 MHz, CD_3OD) spectrum of **6**.

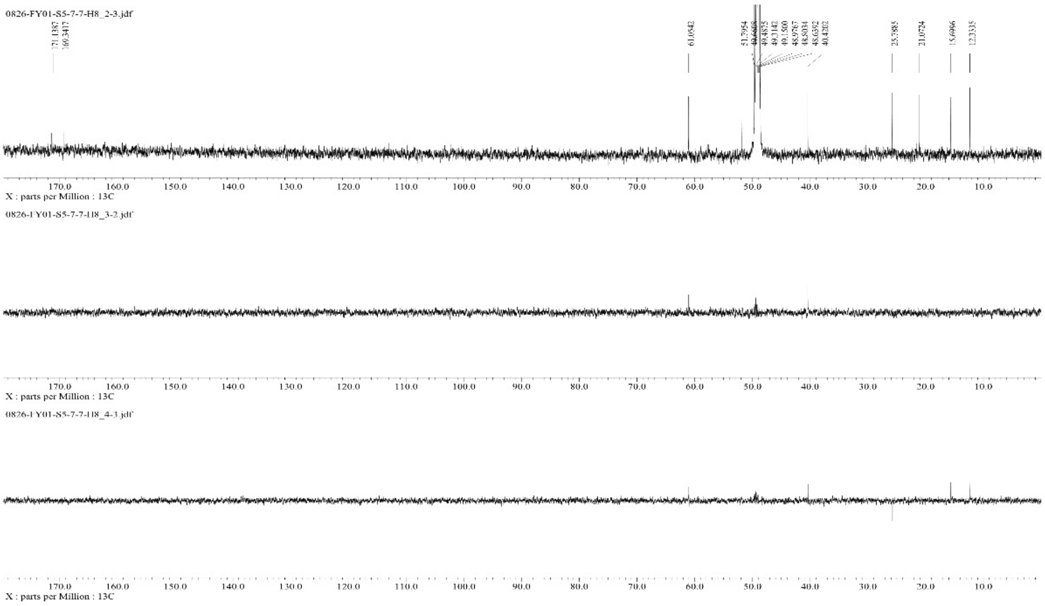

Figure S29. DEPT spectrum of **6**.

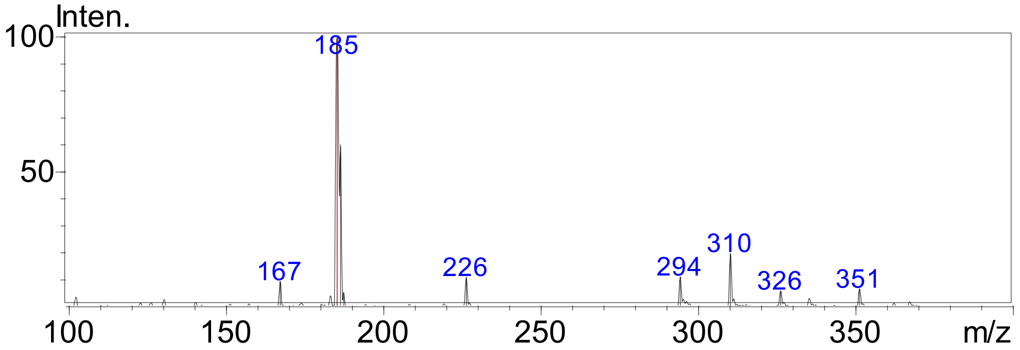

Figure S30. ESIMS spectrum of **7**.

Table S7. 1D NMR (500 MHz, CD_3OD) data of **7**.

Pos.	δ_H (J in Hz) [a]	δ_C (Mult.) [b]
1		
2		171.1 (C)
3	3.96 dd (8.6, 5.1)	54.8 (CH)
4		
5		171.6 (C)
6	4.00 dd (4.9)	52.1 (CH)
7	1.76 m; 1.87 m	45.2 (CH_2)
8	1.66 m	25.5 (CH)
9	0.98 d (6.8)	23.7(CH_3)
10	1.00 d (6.8)	22.3 (CH_3)
11	1.47 d (6.7)	21.0 (CH_3)

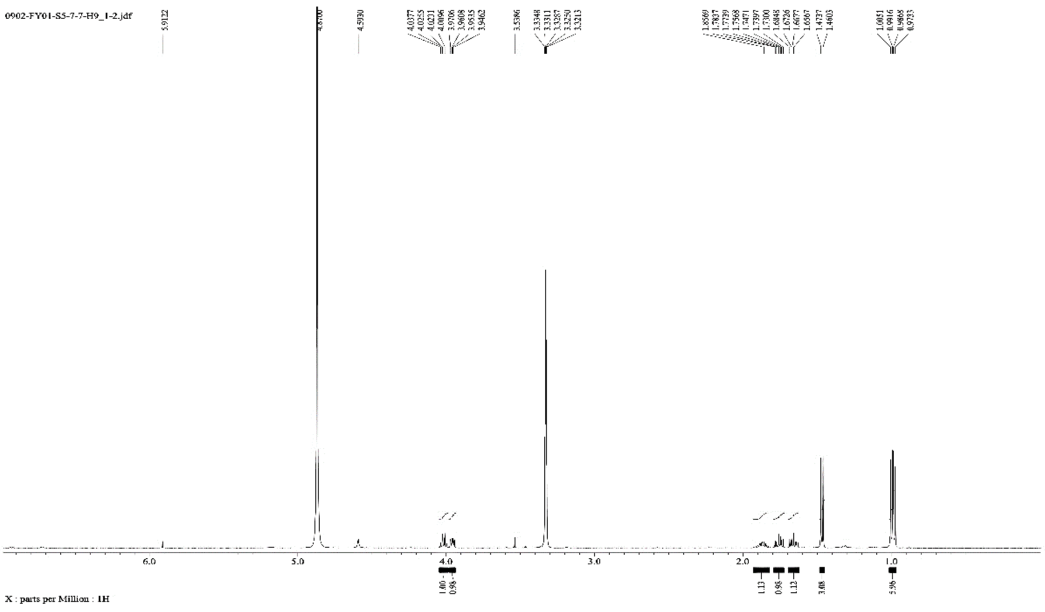

Figure S31. ^1H NMR (500 MHz, CD_3OD) spectrum of **7**.

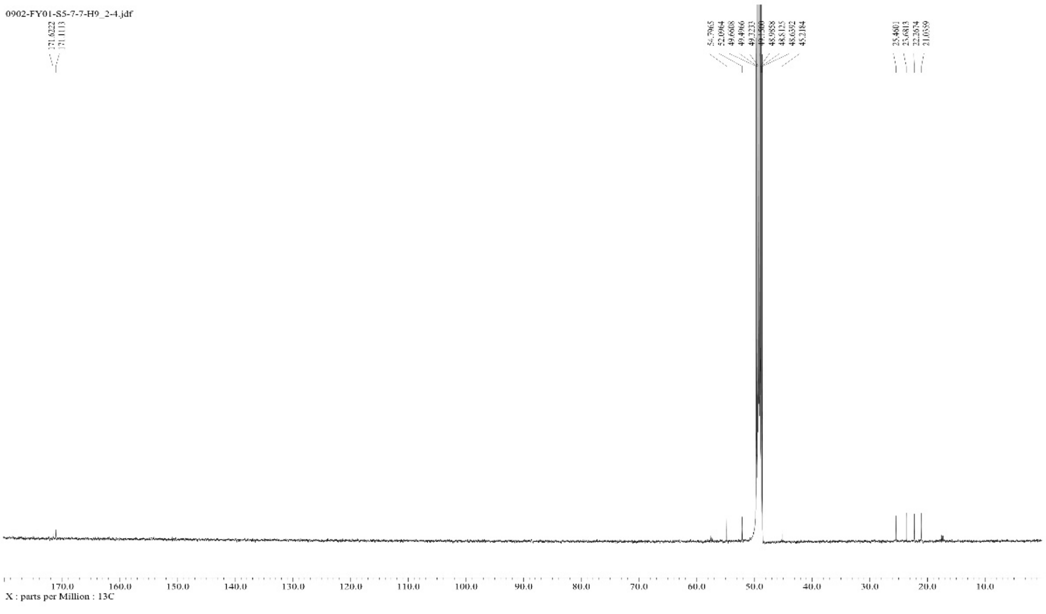

Figure S32. ^{13}C NMR (125 MHz, CD_3OD) spectrum of **7**.

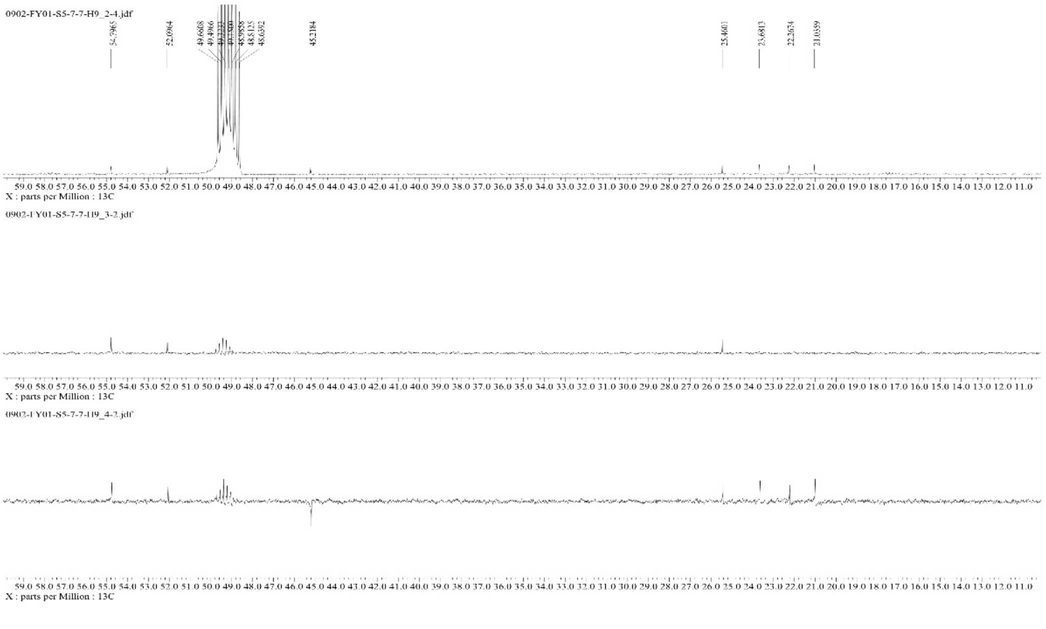

Figure S33. DEPT spectrum of **7**.

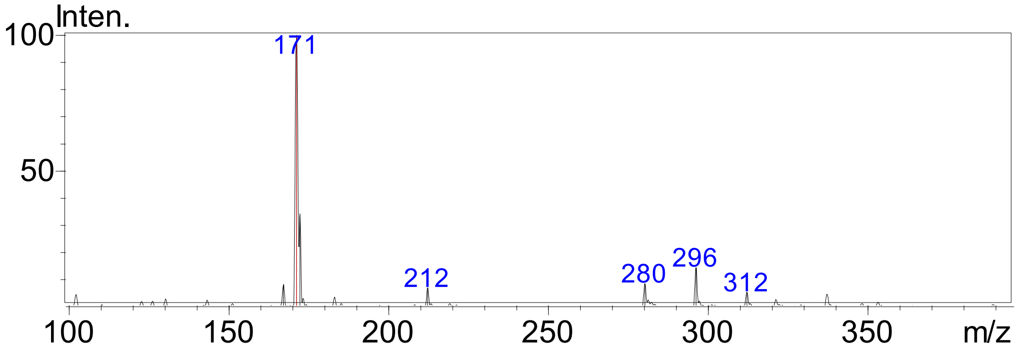

Figure S34. ESIMS spectrum of **8**.

Table S8. 1D NMR (500 MHz, CD_3OD) data of **8**.

Position	δ_H (*J* in Hz) [a]	δ_C (Mult.) [b]
1		
2		171.6 (C)
3	3.91 dd (7.6, 5.8)	55.0 (CH)
4		
5		169.0 (C)
6	3.85 d (17.7); 4.03 d (18.3)	45.4 (CH_2)
7	1.70 m	44.0 (CH_2)
8	1.85 m	25.5 (CH)
9	1.00 d (6.7)	23.5 (CH_3)
10	0.98 d (6.1)	22.3 (CH_3)

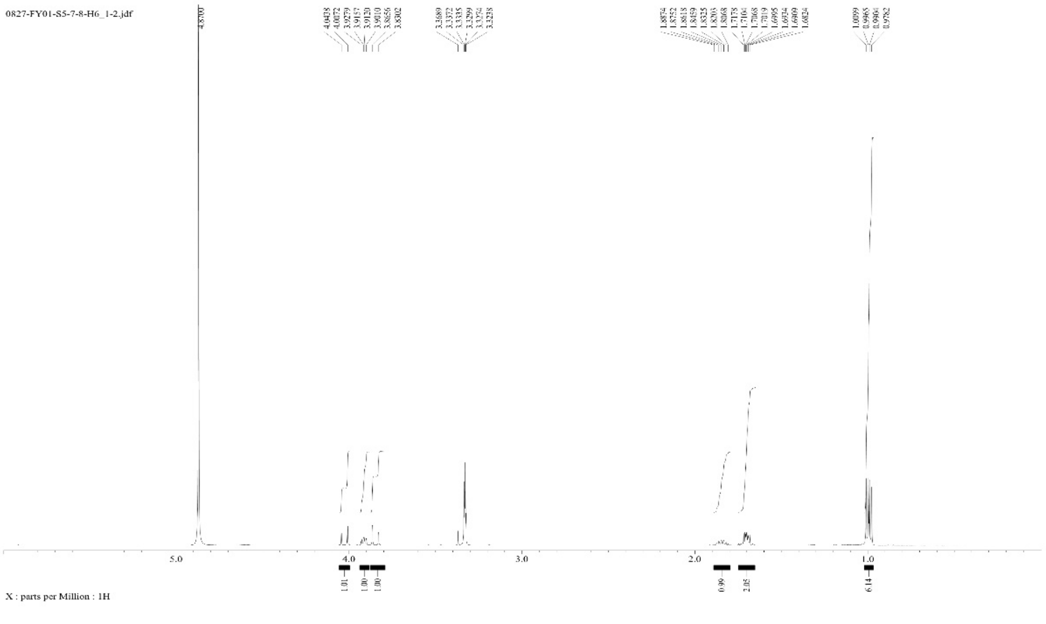

Figure S35. ^{1}H NMR (500 MHz, CD_3OD) spectrum of **8**.

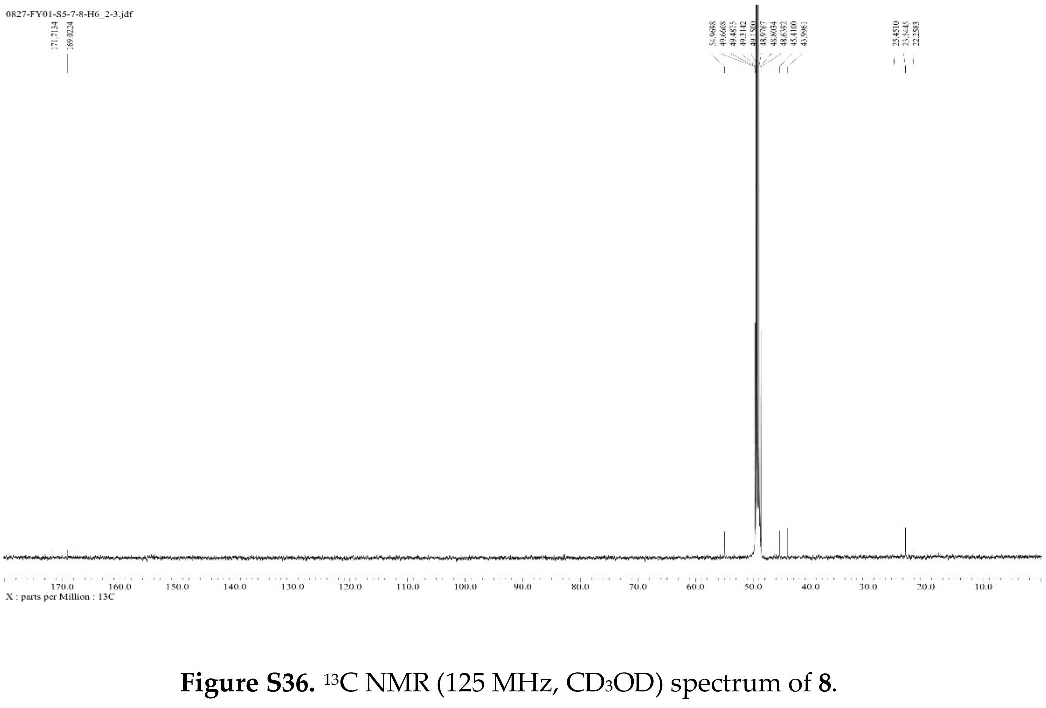

Figure S36. ^{13}C NMR (125 MHz, CD_3OD) spectrum of **8**.

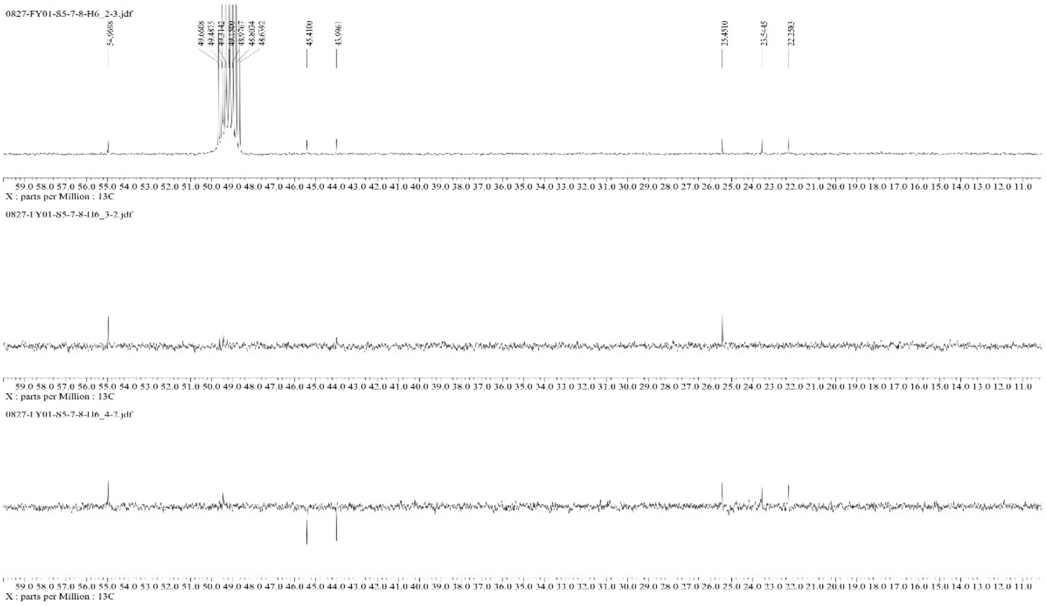

Figure S37. DEPT spectrum of **8**.

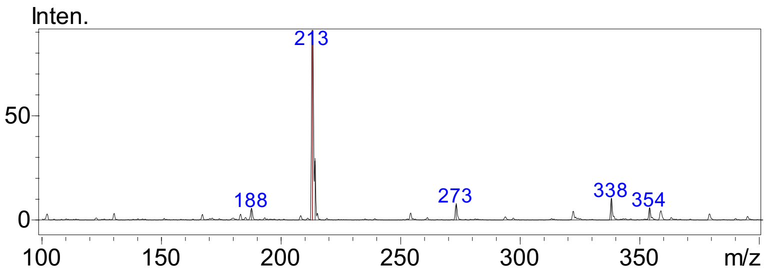

Figure S38. ESIMS of spectrum **9**.

Table S9. 1D NMR (500 MHz, CD_3OD) data of **9**.

Pos.	δ_H (J in Hz) [a]	δ_C (Mult.) [b]
1		
2		167.9 (C)
3	4.08, m	61.6 (CH)
4		
5		173.1 (C)
6	4.49, m	58.9 (CH)
7	2.04, ddd (14.5, 10.3, 3.0)	38.8 (CH_2)
	2.29, dd(13.0, 3.0)	
8	4.46, m	69.0 (CH)
9	3.42, d (13.0)	55.3 (CH_2)
	3.72, dd (13.0, 2.5)	
10	2.49, pd (7.0, 2.5)	29.9 (CH)
11	1.10, d (7.5)	19.0 (CH_3)
12	0.94, d (7.0)	16.8 (CH_3)

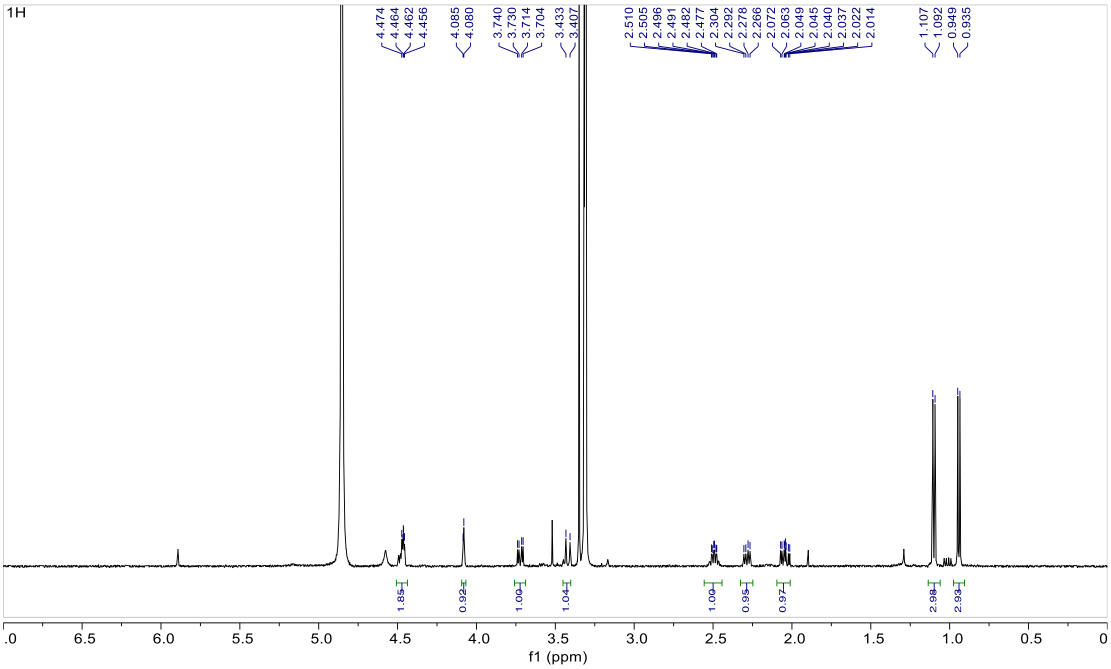

Figure S39. ^{1}H NMR (500 MHz, CD_3OD) spectrum of **9**.

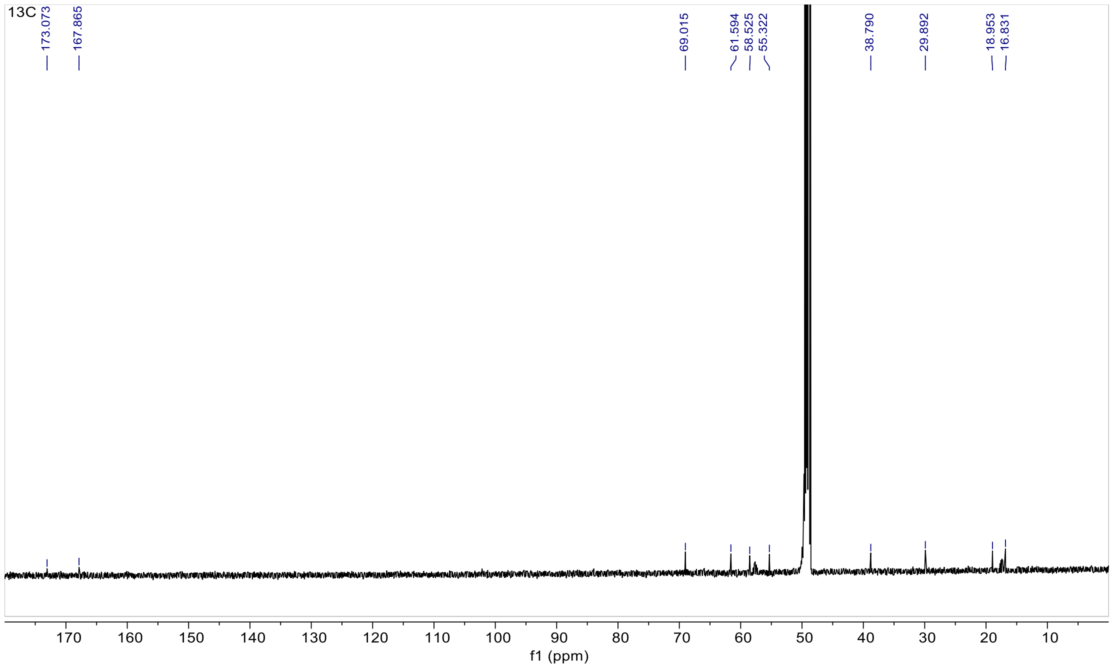

Figure S40. ^{13}C NMR (125 MHz, CD_3OD) spectrum of **9**.

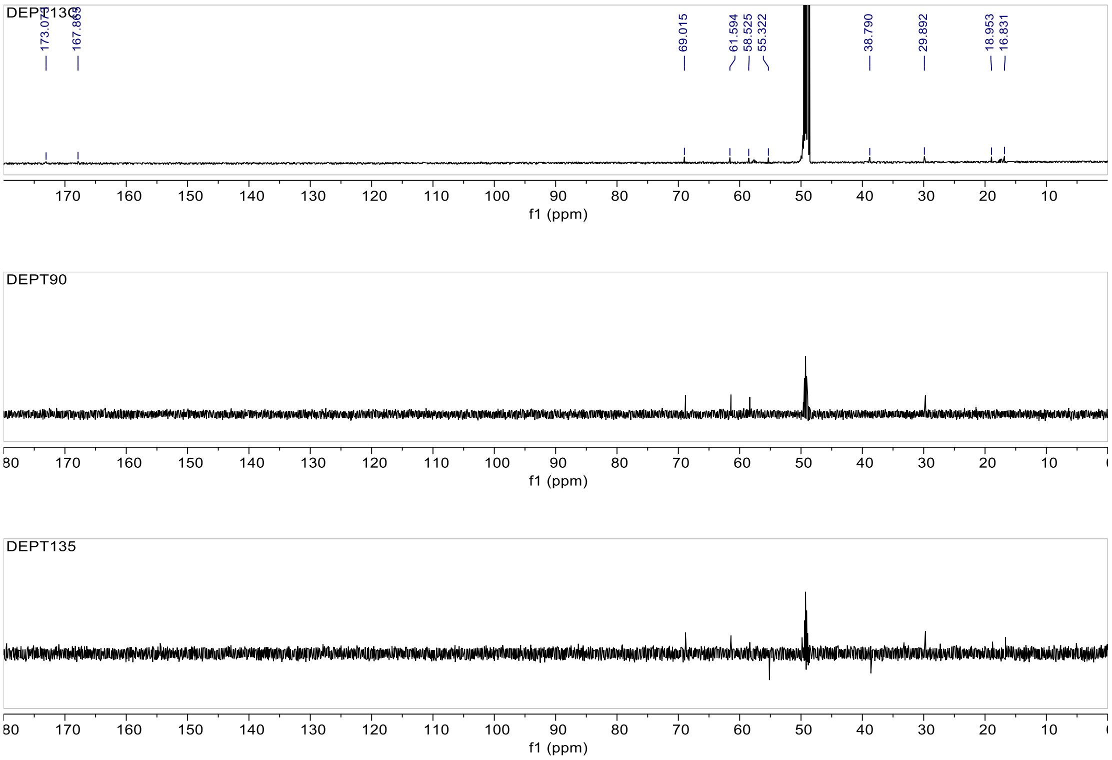

Figure S41. DEPT spectrum of **9**.

Table S10. 1D NMR (500 MHz, CD_3OD) data of **10**.

**Pos.**	**δ_H (J in Hz)** [a]	**δ_C (Mult.)** [b]
1		
2		169.2 (C)
3	4.16 m	54.8 (CH)
4		
5		173.2 (C)
6	4.51 ddd (11.0, 6.4, 1.5)	58.9 (CH)
7	2.27 q (6.6)<br>2.08 ddd (14.3, 10.1, 3.3)	38.3 (CH_2)
8	4.45 t (4.3)	69.3 (CH)
9	3.65 dd (12.8, 2.1)<br>3.43 d (12.8)	55.3 (CH_2)
10	1.93 m<br>1.51 m	39.5 (CH_2)
11	1.89 m	25.8 (CH)
12	0.96 d (3.1)	23.3 (CH_3)
13	0.95 d (2.5)	22.2 (CH_3)

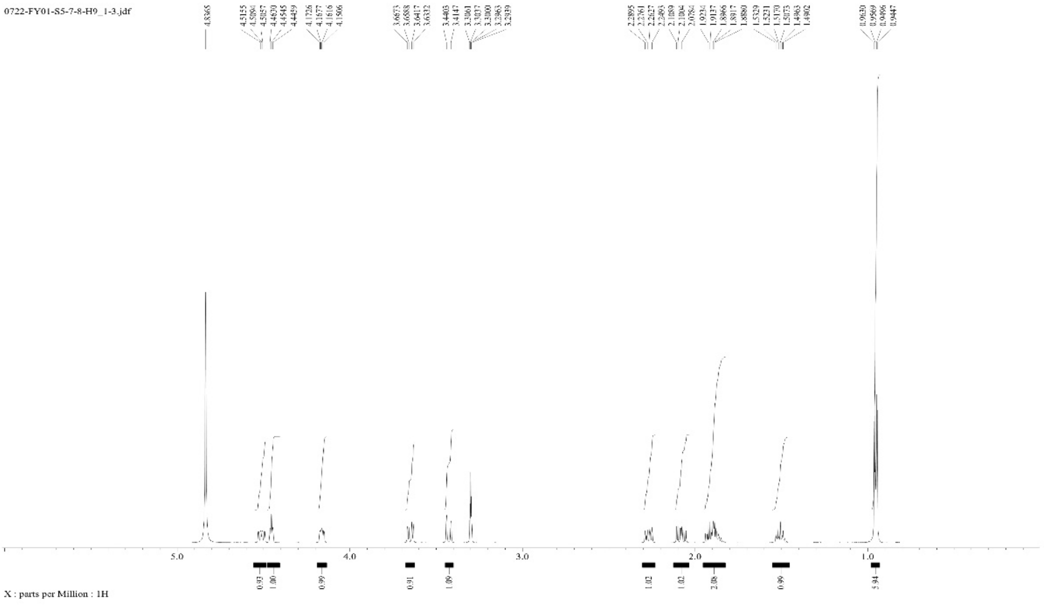

Figure S42. ^{1}H NMR (500 MHz, CD_3OD) spectrum of **10**.

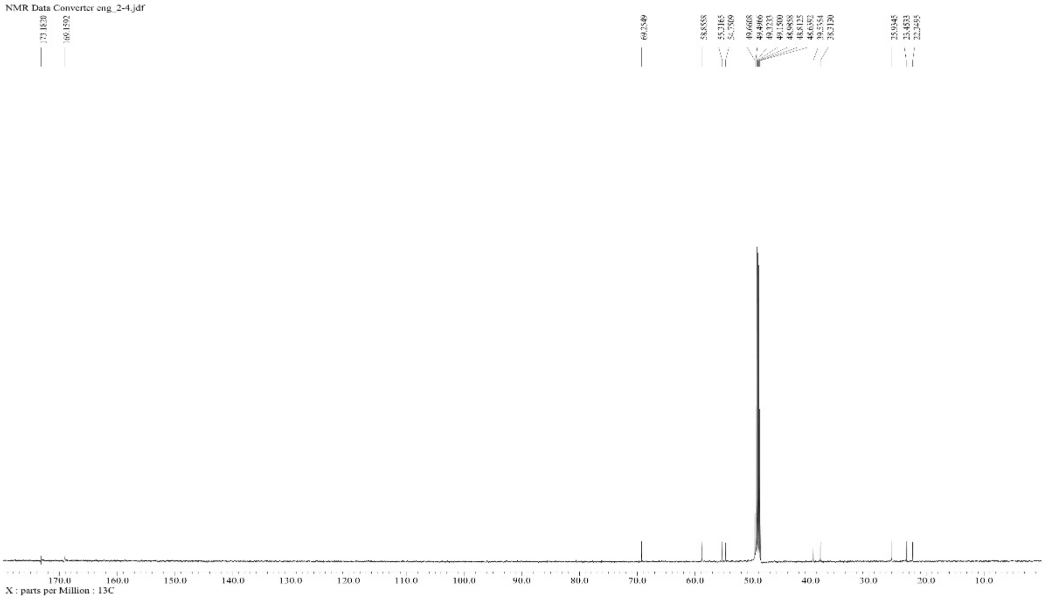

Figure S43. ^{13}C NMR (125 MHz, CD_3OD) spectrum of **10**.

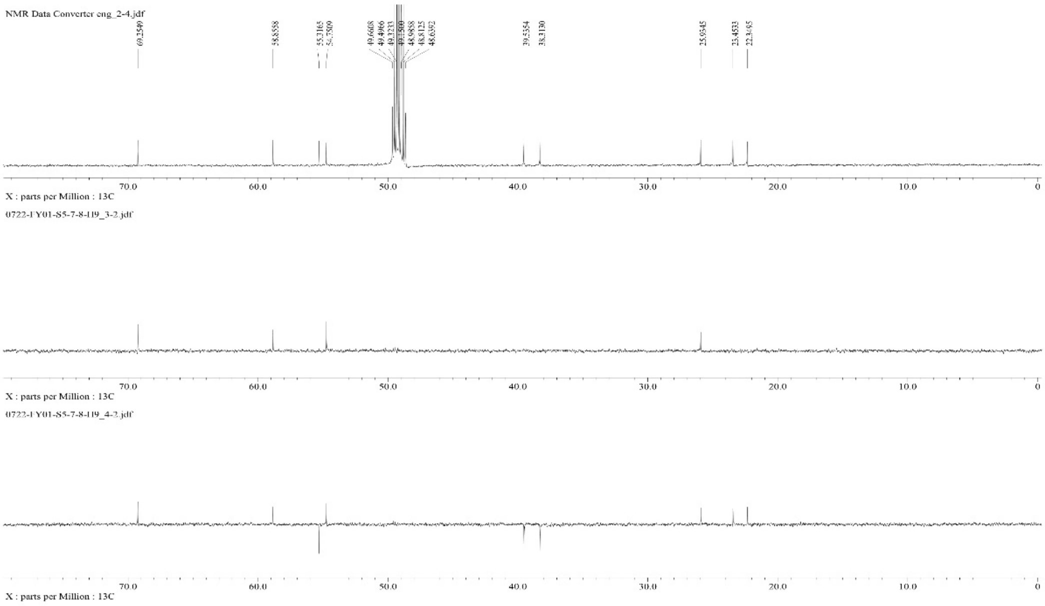

Figure S44. DEPT spectrum of **10**.

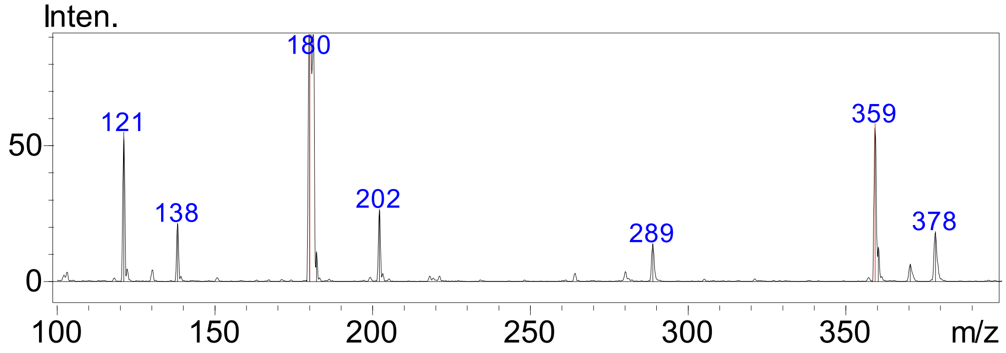

Figure S45. ESIMS spectrum of **11**.

Table S11. 1D NMR (500 MHz, CD_3OD) data of **11**.

Pos.	δ_H (J in Hz)	δ_C (Mult.)
1		155.5 (C)
2	6.95 d (8.6)	129.3 (CH)
2′		
3	6.64 (8.5)	114.8 (CH)
3′		
4		129.8 (C)
5	2.61 t (7.6)	34.3 (CH_2)
6	3.26 m	41.0 (CH_2)
6-OAc	1.83 s	21.3 (CH_3)
		171.8 (C)

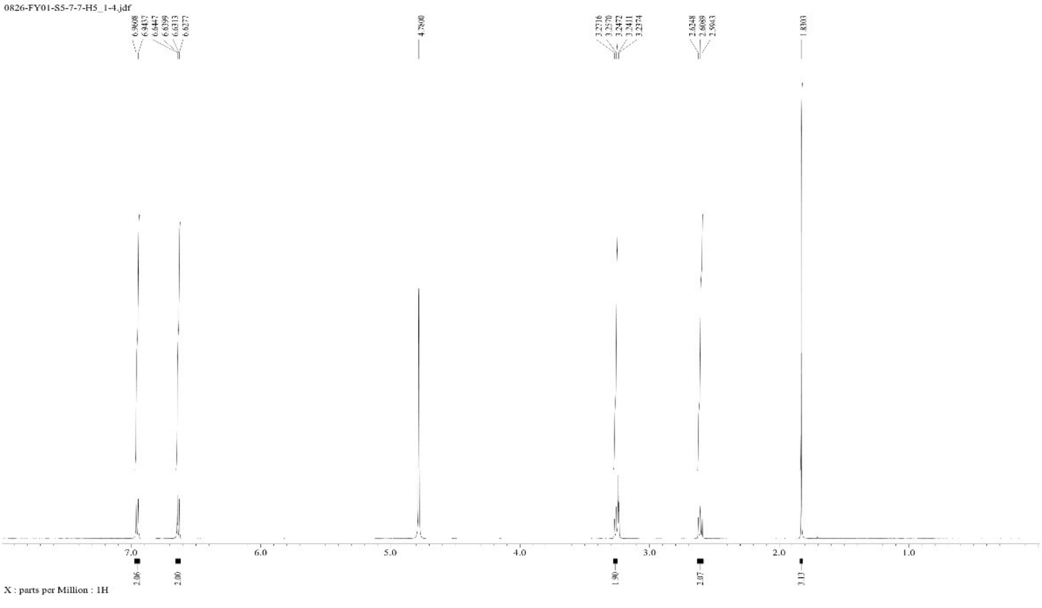

Figure S46. ^{1}H NMR (500 MHz, CD_3OD) spectrum of **11**.

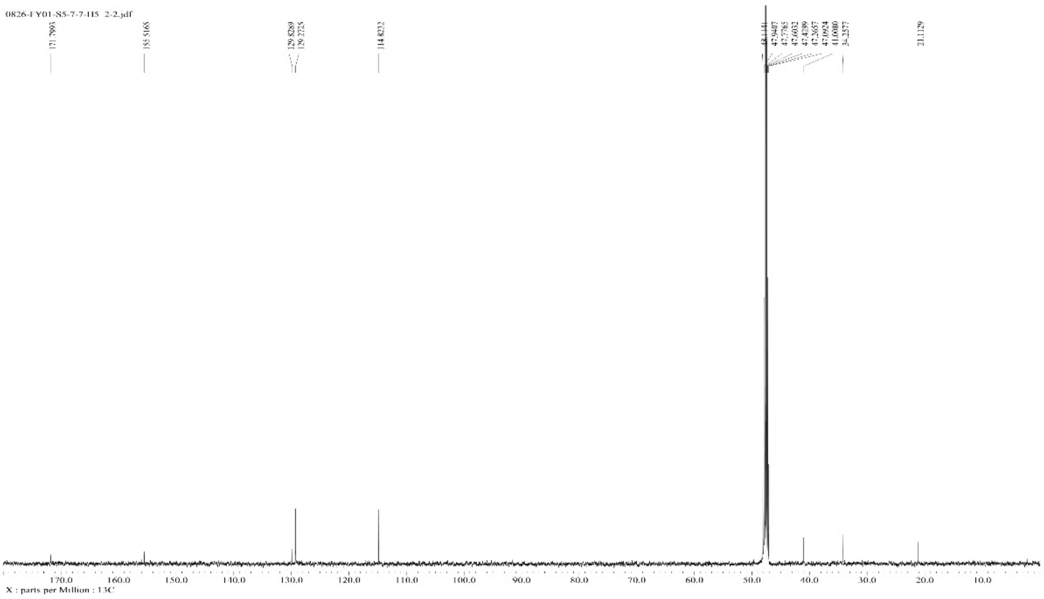

Figure S47. ^{13}C NMR (125 MHz, CD_3OD) spectrum of **11**.

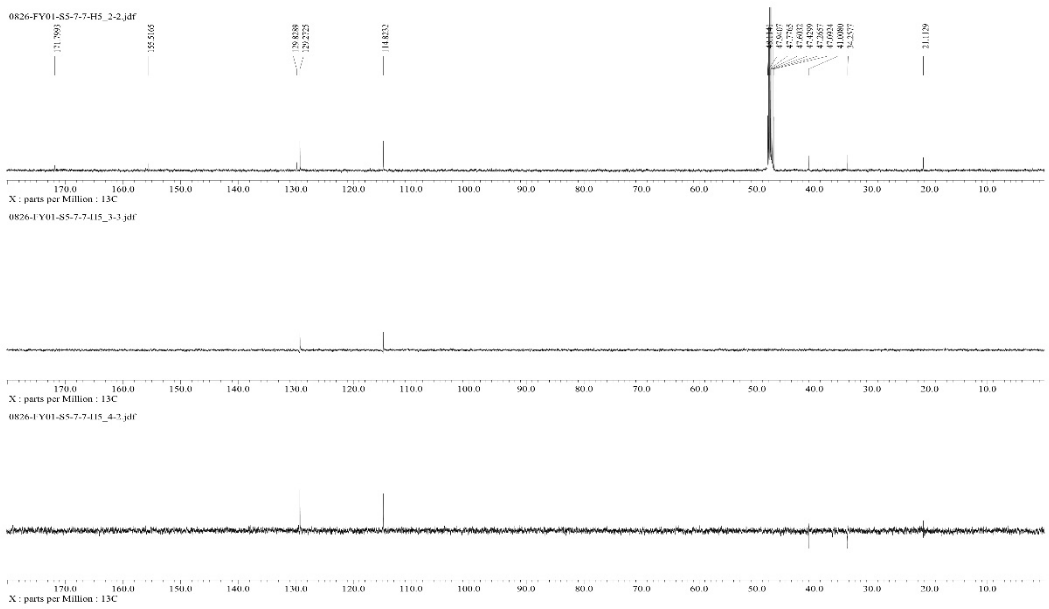

Figure S48. DEPT spectrum of **11**.
